# The efficacy of the benzimidazoles oxfendazole and flubendazole against *Litomosoides sigmodontis* is dependent on the adaptive and innate immune system

**DOI:** 10.3389/fmicb.2023.1213143

**Published:** 2023-06-27

**Authors:** Frederic Risch, Johanna F. Scheunemann, Julia J. Reichwald, Benjamin Lenz, Alexandra Ehrens, Joséphine Gal, Frédéric Fercoq, Marianne Koschel, Martina Fendler, Achim Hoerauf, Coralie Martin, Marc P. Hübner

**Affiliations:** ^1^Institute for Medical Microbiology, Immunology and Parasitology, University Hospital Bonn, Bonn, Germany; ^2^German Center for Infection Research (DZIF), Partner Site Bonn-Cologne, Bonn, Germany; ^3^Unité Molécules de Communication et Adaptation des Microorganismes, Sorbonne Université, Muséum national d’Histoire naturelle, CNRS, Paris, France

**Keywords:** filariae, *Litomosoides sigmodontis*, oxfendazole, flubendazole, macrofilaricide, combination therapy, helminths, benzimidazole

## Abstract

Filarial nematodes can cause debilitating diseases such as lymphatic filariasis and onchocerciasis. Oxfendazole (OXF) is one promising macrofilaricidal candidate with improved oral availability compared to flubendazole (FBZ), and OXF is currently under preparation for phase 2 clinical trials in filariasis patients. This study aimed to investigate the immune system’s role during treatment with OXF and FBZ and explore the potential to boost the treatment efficacy via stimulation of the immune system. Wild type (WT) BALB/c, eosinophil-deficient *ΔdblGata1*, *IL-4r/IL-5^−/−^*, antibody-deficient μMT and B-, T-, NK-cell and ILC-deficient *Rag2/IL-2rγ^−/−^* mice were infected with the rodent filaria *Litomosoides sigmodontis* and treated with an optimal and suboptimal regimen of OXF and FBZ for up to 5 days. In the second part, WT mice were treated for 2–3 days with a combination of OXF and IL-4, IL-5, or IL-33. Treatment of WT mice reduced the adult worm burden by up to 94% (OXF) and 100% (FBZ) compared to vehicle controls. In contrast, treatment efficacy was lower in all immunodeficient strains with a reduction of up to 90% (OXF) and 75% (FBZ) for *ΔdblGata1*, 50 and 92% for *IL-4r/IL-5^−/−^*, 64 and 78% for μMT or 0% for *Rag2/IL-2rγ^−/−^* mice. The effect of OXF on microfilariae and embryogenesis displayed a similar pattern, while FBZ’s ability to prevent microfilaremia was independent of the host’s immune status. Furthermore, flow cytometric analysis revealed strain-and treatment-specific immunological changes. The efficacy of a shortened 3-day treatment of OXF (−33% adult worms vs. vehicle) could be boosted to a 91% worm burden reduction via combination with IL-5, but not IL-4 or IL-33. Our results suggest that various components of the immune system support the filaricidal effect of benzimidazoles *in vivo* and present an opportunity to boost treatment efficacy.

## Introduction

Parasitic filarial nematodes are the causative agents of several diseases such as lymphatic filariasis (*Wuchereria bancrofti*, *Brugia malayi*, and *Brugia timori*), onchocerciasis (*Onchocerca volvulus*), loiasis (*Loa loa*) and mansonelliasis (*Mansonella perstans*, *Mansonella streptocerca*, and *Mansonella ozzardi*). The WHO includes lymphatic filariasis and onchocerciasis in its list of neglected tropical diseases (NTD) (WHO, 2020). Lymphatic filariasis may manifest with symptoms like lymphedema, hydrocele or elephantiasis and is prevalent in 72 countries with an estimated 51.4 million people infected in 2018 (Taylor et al., 2010; WHO, 2020). Patients with onchocerciasis can develop symptoms like dermatitis, visual impairment and blindness (Taylor et al., 2010). Onchocerciasis, also known as river blindness, is responsible for an estimated 1.3 million disability-adjusted life years, with roughly 21 million people infected in sub-Saharan Africa and minor foci in South America (James et al., 2018; WHO, 2020).

Both lymphatic filariasis and onchocerciasis are mainly treated via mass drug administration (MDA) campaigns which consist of (bi-)annual community-wide treatment efforts (WHO, 2020). The current recommendations for MDA of lymphatic filariasis are a combination of ivermectin, albendazole and diethylcarbamazine (Irvine et al., 2017; King et al., 2018; Ehrens et al., 2022a). However, this triple therapy may cause severe adverse events in patients co-infected with loiasis and onchocerciasis (Francis et al., 1985; Boussinesq et al., 2003; Wanji et al., 2018). Thus, in areas co-endemic for onchocerciasis, the WHO recommends treatment with ivermectin alone (WHO, 2020). In areas co-endemic for loiasis, ivermectin and albendazole are used. However, people with high *L. loa* microfilariae loads have to be excluded from the treatment (test-and-not-treat) due to the risk of developing life-threatening adverse events. Moreover, the current treatment strategies have severe limitations as ivermectin, albendazole and diethylcarbamazine are mainly microfilaricidal, i.e., they target and eliminate the filarial progeny, the microfilariae, of the parasites (Geary et al., 2019; Hawryluk, 2020). Thus, the current treatment strategies temporarily interrupt the transmission of the parasite. However, eliminating the diseases will require continuous, community-wide administrations for decades as the adult worms can be reproductively active for at least 5–8 years (Taylor et al., 2010; McLure et al., 2022; Fuller et al., 2023). In addition, as the prevalence of lymphatic filariasis or onchocerciasis decreases, community-wide treatment strategies become less cost-effective, and the transition to more targeted approaches that cure the disease, i.e., clear the adult worms (=macrofilaricidal), rather than prevent transmission become more relevant (Irvine et al., 2017). As a result, the WHO has outlined the development of novel macrofilaricidal compounds or treatment strategies as one of the critical actions to combat onchocerciasis in the 2030 NTD roadmap (WHO, 2020). Potential macrofilaricidal compounds that are currently being tested in phase II clinical trials include flubentylosin (ABBV4083), emodepside, and oxfendazole (OXF) (Ehrens et al., 2022a).

OXF, as well as the related compound flubendazole (FBZ), are two benzimidazoles that selectively target the β-tubulin subunits of nematodes and both compounds have shown macrofilaricidal activities against different filarial species (Lacey, 1990; Geary et al., 2019; Gonzalez et al., 2019). Animal studies have reported on the macrofilaricidal efficacy of FBZ against *Brugia pahangi* and *Litomosoides sigmodontis* (Denham et al., 1979; Surin and Denham, 1990; Hübner et al., 2019). However, FBZ has limited oral bioavailability, and early studies in humans have reported severe side effects such as abscess formations and inflammation after parenteral administrations in humans (Dominguez-Vazquez et al., 1983). Studies that attempted to address the issues of FBZ have resulted in an amorphous solid dispersion formulation of FBZ with increased oral bioavailability (Hübner et al., 2019; Lachau-Durand et al., 2019). However, this formulation had only limited efficacy on adult worms of *Onchocerca ochengi* and *Brugia pahangi*, microfilariae of *B. malayi* and caused genotoxicity in an *in vivo* micronucleus test (Fischer et al., 2019; Lachau-Durand et al., 2019; Sjoberg et al., 2019). OXF, on the other hand, is not only effective after subcutaneous injection but also has improved bioavailability compared to FBZ (Ehrens et al., 2022a). OXF has been used in veterinary medicine as a broad-spectrum anthelmintic for over 30 years (Gonzalez et al., 2019; Hawryluk, 2020). Recent studies have shown that OXF is also effective against adult worms of *Onchocerca gutturosa*, L5 stages of *O. volvulus* and adult worms of the rodent filaria *Litomosoides sigmodontis* but possesses limited to no effect against microfilariae (Hübner et al., 2020). Phase I clinical studies with multiple ascending dosages for OXF have been completed and shown no adverse reactions (Bach et al., 2020). Additional bioavailability studies in humans with an OXF field applicable tablet formulation have been tested through the Helminth Elimination Platform (HELP),^1^ and Phase II clinical trials of OXF as a pan-nematode drug against *O. volvulus*, *L. loa*, *M. perstans*, and the intestinal human whipworm *Trichuris trichiura* through the EU-funded eWHORM project^2^ are currently in preparation (Specht and Keiser, 2022).

Despite these advances in developing macrofilaricidal candidates, additional preclinical research may further improve treatment options. One potential avenue to improve the efficacy of anti-filarial treatment is the combination of chemotherapy with immunostimulatory compounds (Murthy et al., 1992; Rahdar et al., 2020; Silva et al., 2020). Thus, this study aimed to characterize the role of the immune system during anti-filarial treatment with OXF and FBZ using the *L. sigmodontis* rodent model and evaluate the potential of boosting the macrofilaricidal activity of OXF via a combination therapy of OXF with various cytokines.

## Materials and methods

### Animals and natural infection with *Litomosoides sigmodontis*

Six-week old female and male BALB/cJ WT mice were purchased from Janvier Labs, Saint-Berthevin, France. BALB/c *ΔdblGata1*, BALB/c *IL-4r/IL-5^−/−^*, BALB/c μMT, and C57BL/6 *Rag2/IL-2rγ^−/−^* were bred at the animal facility “Haus für Experimentelle Therapie” of the University Hospital Bonn. *ΔdblGata1* and μMT mice were originally obtained from Jackson Laboratory (Bar Harbor, United States), *IL-4r/IL-5^−/−^* from Prof. Dr. Klaus Matthaei (Matthaei, Stem Cell & Gene Targeting Laboratory, ANU College of Medicine, Biology and Environment, Canberra, Australia) and *Rag2/IL-2rγ^−/−^* from Taconic Biosciences Inc. (Cologne, Germany).

For the experiments, mice were housed in individually ventilated cages with unlimited access to food and water and a 12-h day/night cycle within the animal facility at the Institute for Medical Microbiology, Immunology and Parasitology (IMMIP), University Hospital Bonn. All animal experiments were performed according to EU Directive 2010/63/EU and approved by the appropriate state authorities Landesamt für Natur-, Umwelt- und Verbraucherschutz, Recklinghausen, Germany (AZ: 84–02.04.2015.A507, 81–02.04.2020.A244, 81–02.05.40.18.057).

Six to twenty week old male and female mice were naturally infected with *L. sigmodontis* via exposure to the tropical rat mite, *Ornithonyssus bacoti*, carrying the infective L3 larvae as described elsewhere (Reichwald et al., 2022). In short, mice were placed in cages with bedding material containing the mites. After 24 h, the bedding material containing the mites was removed, and the cages were placed on top of a plastic tub with disinfectant below the cages and no direct contact with the mice. After an additional 24 h, mice were moved into standard cages, and the bedding material was exchanged daily for 5 days to remove any remaining mites.

### Treatment

For the OXF treatment, a commercially available formulation of OXF (Dolthene) was used and dissolved in corn oil (Sigma-Aldrich, St. Louis, United States). Vehicle controls received only corn oil. For all experiments, treatment with OXF was performed orally, twice per day, 8 h apart starting 35 days post infection (dpi) after the development of adult worms (28–35 dpi) but before the onset of microfilaremia (50–56 dpi) (Hübner et al., 2009). For the experiments with immunodeficient strains, mice received either 5 or 12.5 mg/kg OXF in a total volume of 5 mL/kg per treatment for 5 days (see Supplementary Table S1 for a detailed breakdown of treatments in immunodeficient mice) (Hübner et al., 2020). For the 3-day combination therapy, mice received 12.5 mg/kg OXF for 3 or 5 days, and for the 2-day combination therapy, mice received either 12.5 or 25 mg/kg OXF per treatment for 2 or 5 days. For the 3-day combination therapy, 2 μg of interleukin-4 (IL-4), IL-5 or IL-33 were given intranasally in a volume of 10 μL under short anesthesia induced with 2% isoflurane (AbbVie, Wiesbaden, Germany) once per day for 3 days (Johansson et al., 2018; Beckert et al., 2020). For the 2-day combination therapy, 2 μg of IL-5 was given intranasally in a volume of 10 μL under short anesthesia induced with 2% isoflurane (AbbVie, Wiesbaden, Germany) once per day for 2 days.

For the FBZ treatment, FBZ (Sigma-Aldrich, St. Louis, United States) was dissolved in distilled water with 0.1% v/v tween80 (Sigma-Aldrich, St. Louis, United States) and 0.5% w/v hydroxyethyl cellulose (Sigma-Aldrich, St. Louis, United States). Vehicle controls received only distilled water with 0.1% v/v tween80 and 0.5% w/v hydroxyethyl cellulose. Treatment was performed 35 dpi for 2 or 5 days once per day via subcutaneous injections (Hübner et al., 2019). All mice received 2 mg/kg FBZ in a volume of 5 mL/kg per treatment (see Supplementary Table S1 for a detailed breakdown of treatments in immunodeficient mice).

### Parasite recovery and quantification

Necropsies were performed to quantify the adult worm burden and the number of microfilariae in the peripheral blood at 70 dpi. Mice were euthanized with an overdose of isoflurane, and adult worms were isolated via lavage of the thoracic cavity with 8–10 mL PBS (Thermo Fisher Scientific, Waltham, United States). Isolated worms were counted and the gender was determined. To quantify the microfilariae burden at 56 and 70 dpi, 50 μL of peripheral blood was drawn from the facial vein into EDTA tubes (Sarstedt AG & Co. KG, Nümbrecht, Germany) and incubated with 950 μL red blood cell lysis buffer (Thermo Fisher Scientific, Waltham, United States) for 10 min at room temperature (RT). The blood was centrifuged at 400 × g for 5 min at RT, the supernatant was discarded, and microfilariae in the pellet were counted with a bright-field microscope (Zeiss, Göttingen, Germany) as previously described (Reichwald et al., 2022).

To quantify the embryonal stages, intact, female adult worms were fixed with 4% formaldehyde (Sigma-Aldrich, St. Louis, United States) in PBS (Thermo Fisher Scientific, Waltham, United States) for 24 h and then stored in 60% v/v ethanol in distilled water at RT until further analysis. Worms were then transferred into 1.5 mL Eppendorf tubes (Eppendorf SE, Hamburg, Germany) containing 100 μL Hinkelmann solution (0.5% eosin yellow, 0.5% phenol, 0.185% formaldehyde in distilled water) and crushed with a mortar. Embryonal stages [oocyte, morula, pretzel, stretched microfilaria, degenerated early stage (altered oocyte/morula) and degenerated late stage (altered pretzel/microfilaria)] were then quantified with a bright-field microscope (Zeiss, Göttingen, Germany) as previously described (Ziewer et al., 2012).

### Histology

Lungs from *L. sigmodontis*-infected WT BALB/c mice (*n* = 6–7 per group) were inflated and fixed twice in 4% formalin for 24 h each. Lungs were then dehydrated in ethanol baths of increasing concentrations from 70 to 100% and placed in toluene before paraffin embedding. All sections were cut deep enough to see the main bronchi and perivascular adventitial spaces (PVS). Seven-micron-thick serial sections were prepared and various stainings were performed. (i) Hematoxylin and eosin staining was used to visualize lung structure and performed as follows: Sections were incubated with Mayer’s hematoxylin solution for 5 min, rinsed with tap water for 20 s and then incubated with 1% eosin solution for 1 min. (ii) Alcian Blue—Periodic Acid Schiff (AB-PAS) staining to visualize mucus-producing cells was performed as follows: Sections were incubated with 1% Alcian blue in 3% acetic acid for 20 min, rinsed with tap water and distilled water for 2 min each, incubated with 0.5% periodic acid for 5 min and again rinsed with distilled water. Next, sections were counterstained with Schiff’s reagent for 10 min, rinsed with tap water for 5 min and stained with hematoxylin for 1 min. Finally, sections were rinsed again with tap water for 2 min and then differentiated with acid alcohol. (iii) Luxol Fast Blue staining to visualize eosinophils was performed as follows: Sections were incubated with Luxol blue for 20 min, rinsed with running tap water for 2 min and then counterstained with Mayer’s hematoxylin for 10 s. The number of Luxol blue positive eosinophils was determined in 15 fields of view with an ×100 objective using Olympus BH2. The other sections were scanned at the MNHN light microscopy facility (CeMIM, Centre de Microscopie et d’IMagerie numerique, MNHN Paris) with a NanoZoomer S60 digital slide scanner (Hamamatsu) and images were analyzed with QuPath 0.3 software (Bankhead et al., 2017). For cell infiltration in PVS, 3–5 PVS areas per mouse were segmented manually, Hematoxylin positive nucleus detection was done using the “Cell detection” tool in QuPath and results were expressed as “number of nuclei/mm^2^ of PVS.” Minimal thickness of bronchial arteries (in micrometer) was measured from 3 to 6 arteries of similar diameter per mouse.

### Preparation of organs for flow cytometry analysis

The thoracic cavity lavage was performed with 8–10 mL PBS. The lavage was centrifuged at 400 × g for 5 min at 4°C and the supernatant was discarded. The pellet was resuspended in 1 mL red blood cell lysis buffer (Thermo Fisher Scientific, Waltham, United States). Cells were then washed with PBS containing 1% v/v FCS (PAN Biotech, Aidenbach, Germany) and 2 mM EDTA (Carl Rohe, Karlsruhe, Germany), resuspended in PBS containing 1% v/v FCS and 2 mM EDTA and counted with a CasyR TT Cell Counter (Schärfe Systems, Reutlingen, Germany). 1 × 10^6^ cells were used for flow cytometric analysis.

To isolate splenocytes, spleens were perfused with 3 mL of a 0.5 mg/mL collagenase VIII solution (Roche, Basel, Switzerland), cut into small pieces and incubated at 37°C for 30 min on a shaker with 200 rpm. Five milliliter PBS containing 1% v/v FCS and 2 mM EDTA were then added and the minced spleens were pushed through a 70 μm metal sieve to generate single-cell suspensions. Cells were then centrifuged at 400 × g for 5 min at 4°C and the supernatant was discarded. Red blood cell lysis was performed by incubating the pellet in 1 mL red blood cell lysis buffer (Thermo Fisher Scientific, Waltham, United States). Cells were then washed with PBS containing 1% v/v FCS (PAN Biotech, Aidenbach, Germany) and 2 mM EDTA (Carl Rohe, Karlsruhe, Germany), resuspended in PBS containing 1% v/v FCS and 2 mM EDTA and counted with a CasyR TT Cell Counter (Schärfe Systems, Reutlingen, Germany). 1 × 10^6^ cells were used for flow cytometric analysis.

### Flow cytometry

Cells from the thoracic cavity and spleen were analyzed with one surface staining to identify lymphoid cells and two intracellular stainings to identify T helper cell subsets and myeloid cells. For the surface staining, cells were incubated for 20 min on ice in a mastermix in PBS with 1% v/v FCS, 0.1% v/v rat IgG (Sigma-Aldrich, St. Louis, United States) containing the following antibodies: anti-CD3 (Al700, clone GK1.5, BioLegend), anti-CD4 (BV605, clone RM4-5, BioLegend), anti-CD5 (PerCP Cy5.5, 53–7.3, BioLegend), anti-CD8 (PE, 53–6.7, BioLegend), and anti-CD19 (APC, eBio1D3, Thermo Fisher Scientific). Cells were then washed twice with PBS containing 1% v/v FCS, resuspended in 200 μL PBS containing 1% v/v FCS and 2 mM EDTA and filtered through 70 μm gauze (Labomedic, Bonn, Germany) before measurement with a CytoFLEX S (Beckmann Coulter, Brea, United States) and further analysis with FlowJo V10 (FlowJo, Ashland, United States).

For the intracellular staining, cells were incubated in a fixation/permeabilization buffer (Thermo Fisher Scientific, Waltham, United States) for 20 min at RT. Cells were then centrifuged at 400 × g for 5 min at 4°C and incubated overnight in PBS containing 1% w/v bovine serum albumin fraction V (PAA Laboratories, Cölbe, Germany) and 0.1% v/v rat IgG (Sigma-Aldrich, St. Louis, United States) at 4°C. The following day, cells were centrifuged at 400 × g for 5 min at 4°C and incubated in a permeabilization buffer (Thermo Fisher Scientific, Waltham, United States) for 20 min at RT. Cells were again centrifuged at 400 × g for 5 min at 4°C and stained with master mixes in PBS containing 1% w/v bovine serum albumin fraction V (PAA Laboratories, Cölbe, Germany) and the following antibodies for 45 min at 4°C in the dark: anti-CD3 (BV510, clone 145-2C11, BioLegend), anti-CD4 (Al700, clone 17A2, BioLegend), anti-CD8 (PerCP Cy5.5, clone 53–6.7, BioLegend), anti-CD11b (Al700, clone M1/70, BioLegend), anti-CD11c (BV605, clone N418, BioLegend), anti-CD25 (BV421, PC61, BioLegend), anti-GATA-3 (Al488, clone 16E10A23, BioLegend), anti-FOXP3 (PE-Cy7, FJK-16S, Thermo Fisher Scientific), anti-I-ab (BV421, M5/114.15.2, BioLegend), anti-Ly6C (APC-Cy7, clone HK1.4, BioLegend), anti-Ly6G (PE-Cy7, clone 1A8, BioLegend), anti-RELM-α (APC, clone DS8RELM, Thermo Fisher Scientific), anti-RORγT (PE, clone AFKJS-9, Thermo Fisher Scientific), Siglec-F (PE, clone E50-2440, BD Biosciences) and anti-T-bet (APC, clone 4B10, BioLegend). Cells were then washed twice with PBS containing 1% v/v FCS, resuspended in 200 μL PBS containing 1% v/v FCS and 2 mM EDTA and filtered through 70 μm gauze (Labomedic, Bonn, Germany) before measurement with a CytoFLEX S (Beckmann Coulter, Brea, United States) and further analysis with FlowJo V10 (FlowJo, Ashland, United States).

### Statistical analysis

Statistical analysis was performed with GraphPad Prism software version 8/9 (GraphPad Software, San Diego, United States). The choice of statistical analysis was based on sample size and distribution of samples. Normality was assessed via the Shapiro–Wilk test. For normally distributed data, a One-Way ANOVA with Dunnet’s multiple comparison test was performed. For non-parametric data, the Kruskal-Wallis test followed by Dunn’s multiple comparisons test was used to assess significant differences between 3 or more groups or the Mann–Whitney-U test for differences between two groups. Data for adult worms, microfilariae, immune cells and histological analysis are shown as median with interquartile range. Data for embryonal stages in stacked bar graphs are shown as mean with standard error of mean. *p* < 0.05 were considered to be significant.

## Results

### Treatment efficacy of oxfendazole against *Litomosoides sigmodontis* is reduced in immunocompromised mice

To characterize the role of the immune system during anti-filarial treatment, we naturally infected WT BALB/c mice and several immunodeficient strains, i.e., *ΔdblGata1*, *IL-4r/IL-5^−/−^*, μMT, and *Rag2/IL-2rγ^−/−^,* with *Litomosoides sigmodontis* and treated them orally with 5 or 12.5 mg/kg OXF twice per day for 5 days after the development of adult worms (35 dpi) but before the appearance of microfilariae at 50–56 dpi. Necropsies were performed 70 dpi to quantify worm burden and immunological changes.

**Figure 1:**
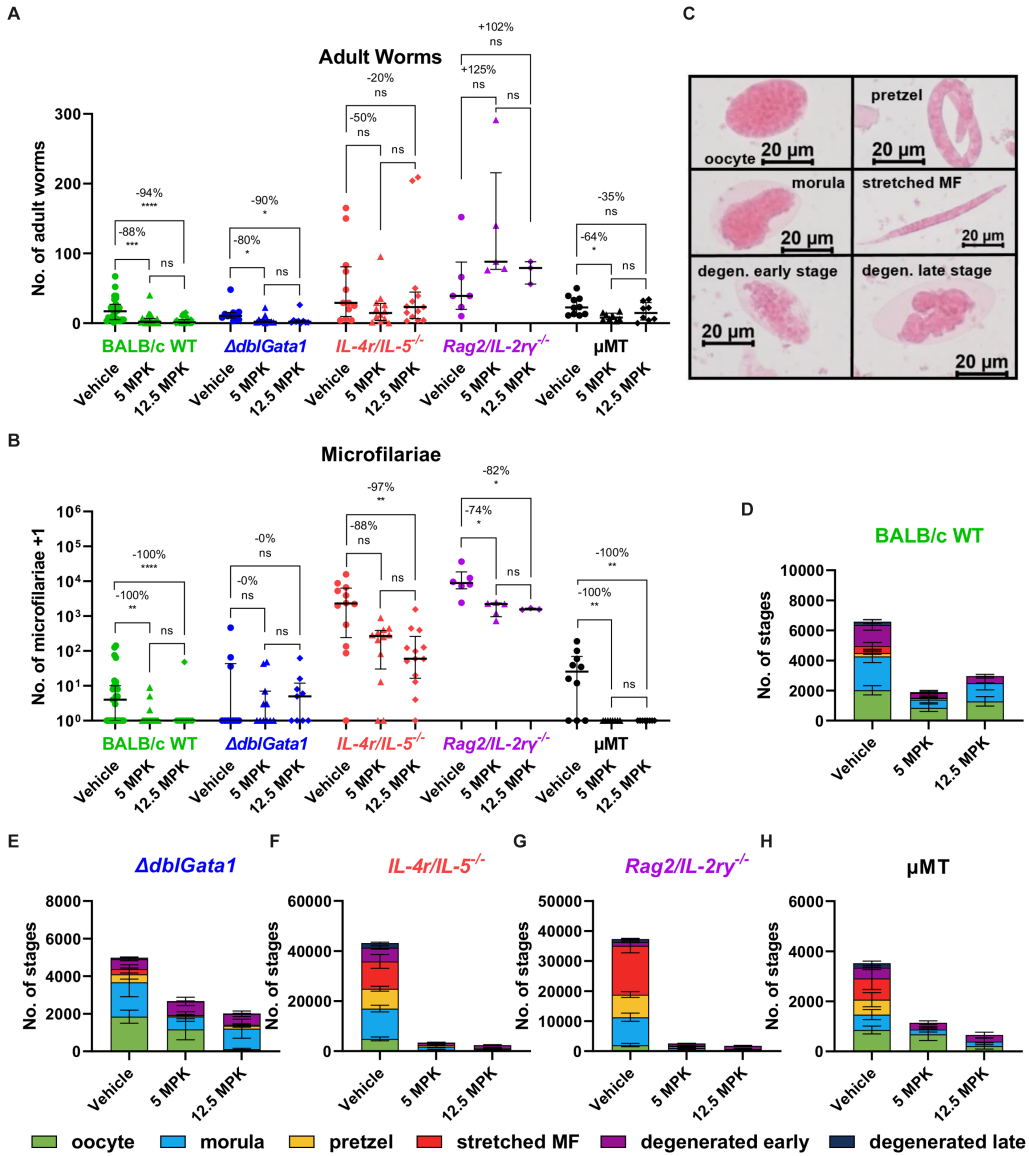
Reduced treatment efficacy of oxfendazole in immunodeficient mice. **(A–H)** Indicated mouse strains were naturally infected with *Litomosoides sigmodontis* and treated orally with 5 or 12.5 mg/kg oxfendazole twice per day for 5 days starting 35 days after the infection. Necropsies were performed 70 days after the infection. **(A)** Adult worm burden. **(B)** Microfilariae per 50 μL peripheral blood +1. **(C)** Representative images of embryonal stages. **(D–H)** Average number of embryonal stages per female worm in **(D)** BALB/c (green), **(E)** *ΔdblGata1* (blue), **(F)** *IL-4r/IL-5^−/−^* (red), **(G)** *Rag2/IL-2rγ^−/−^* (purple), and **(H)** μMT mice (black). **(A,B)** Data shown as median with interquartile range. Numbers show reduction of median in comparison to corresponding vehicle control. **(D–H)** Data shown as mean ± SEM. **(A,B,D–H)** Data for BALB/c pooled from 6 experiments, *IL-4r/IL-5^−/−^* pooled from 3 experiments, *ΔdblGata1*, *Rag2/IL-2rγ^−/−^* and μMT pooled from 2 experiments. Statistical analysis using Kruskal-Wallis with Dunn’s *post-hoc* test, *p < 0.05, **p < 0.01, ***p < 0.001, ****p < 0.0001.

Treatment with both 5 and 12.5 mg/kg OXF led to a statistically significant reduction of the median adult worm burden only in BALB/c WT (88 and 94% respectively) and *ΔdblGata1* mice (80 and 90%) (Figure 1A). The reduction of the adult worm burden in the other strains was either not statistically significant with a median reduction of up to 50% or only statistically significant for one treatment condition, i.e., a reduction of 64% in μMT mice after treatment with 5 mg/kg OXF (Figure 1A). Interestingly, the strain with the most severe immunodeficiency (*Rag2/IL-2rγ^−/−^*) displayed no reduction of the adult worm burden after treatment at all (Figure 1A). The effect of the treatment on the numbers of microfilariae in the peripheral blood presented a different picture (Figure 1B). Here we could observe a median reduction of 100% in BALB/c WT mice as well as the mature B cell-and antibody-deficient μMT mice whereas all other strains displayed no (*ΔdblGata1*) or only impaired effects on microfilariae numbers (*IL-4r/IL-5^−/−^, Rag2/IL-2rγ^−/−^*) (Figure 1B). Accordingly, treatment in immunodeficient mice led to fewer adult worm or microfilariae-negative animals (with the exception of μMT mice) compared to the treatment in BALB/c WT animals (Table 1). Importantly, the treatment was performed 35–39 dpi while microfilariae are only detectable in the peripheral blood after 49–56 days indicating that the effect of OXF was based on changes in the embryogenesis of adult worms rather than a direct effect against microfilariae.

**Table 1:** Clearance of adult worms and microfilariae after OXF treatment.

	Adult worm negative mice			Microfilariae negative mice		
	Vehicle	5 mg/kg OXF	12.5 mg/kg OXF	Vehicle	5 mg/kg OXF	12.5 mg/kg OXF
BALB/c	0.0%	23.8%	44.4%	44.4%	85.7%	96.2%
	(0/27)	(5/21)	(12/27)	(12/27)	(18/21)	(26/27)
*ΔdblGata1*	0.0%	9.0%	11.1%	70.0%	54.5%	44.4%
	(0/10)	(1/11)	(1/9)	(7/10)	(6/11)	(4/9)
*IL-4r/* *IL-5^−/−^*	0.0%	8.3%	0.0%	8.3%	16.60%	7.6%
	(0/12)	(1/12)	(0/13)	(1/12)	(2/12)	(1/13)
*Rag2/* *IL-2rγ^−/−^*	0.0%	0.0%	0.0%	0.0%	0.0%	0.0%
	(0/6)	(0/5)	(0/3)	(0/6)	(0/5)	(0/3)
μMT	0.0%	0.0%	12.5%	30.0%	100%	100%
	(0/10)	(0/8)	(1/8)	(3/10)	(8/8)	(8/8)

BALB/c WT mice and immunodeficient strains (*ΔdblGata1*, *IL-4r/IL-5^−/−^*, μMT, and *Rag2/IL-2rγ^−/−^*) were naturally infected with *Litomosoides sigmodontis* and treated with 5 or 12.5 mg/kg OXF twice per day for 5 days starting 35 dpi. Number of adult worms in the thoracic cavity and number of microfilariae per 50 μL blood were quantified 70 dpi. Data is pooled from 2 to 6 experiments. Shown is the number and frequency of animals that were free of adult worms and microfilariae at 70 dpi.

To further assess the fertility of female worms, we analyzed drug effects on embryogenesis. Therefore, female worms were homogenized and stained with Hinkelmann solution and different embryonal stages, i.e., oocyte, morulae, pretzel, stretched microfilariae as well as degenerated early (oocyte, morulae) and degenerated late stages (pretzel, microfilariae), were counted with a bright field microscope (Figure 1C). Here, *IL-4r/IL-5^−/−^* and *Rag2/IL-2rγ^−/−^* mice had significantly more embryonal stages in the untreated mice than the other strains and treatment with OXF led to a substantial reduction of embryonal stages in all tested strains (Figures 1D–H). In line with the microfilariae numbers (Figure 1B), a closer examination of the treatment effect revealed a nearly complete elimination of late stages (<10 pretzel or stretched microfilariae per worm) only in BALB/c WT and μMT mice but not the other immunodeficient strains (Supplementary Table S2). Taken together, the macrofilaricidal efficacy of OXF appears to be influenced by the innate and adaptive immune system with a more substantial decrease in effectiveness in more severe immunodeficient strains. In contrast, the effect on microfilaremia appears to be influenced by components of innate and adaptive immunity but independent of mature B cells or antibodies and mediated via a reduction in fertility of adult female worms.

### Macrofilaricidal efficacy of flubendazole is reduced in immunocompromised mice

Next, we analyzed the efficacy of FBZ in immunodeficient mice by treating mice that were naturally infected with *L. sigmodontis* with 2 mg/kg FBZ subcutaneously for 2 or 5 days once per day starting treatment at 35 dpi. Necropsies were again performed 70 dpi to investigate the worm burden and immunological changes.

**Figure 2:**
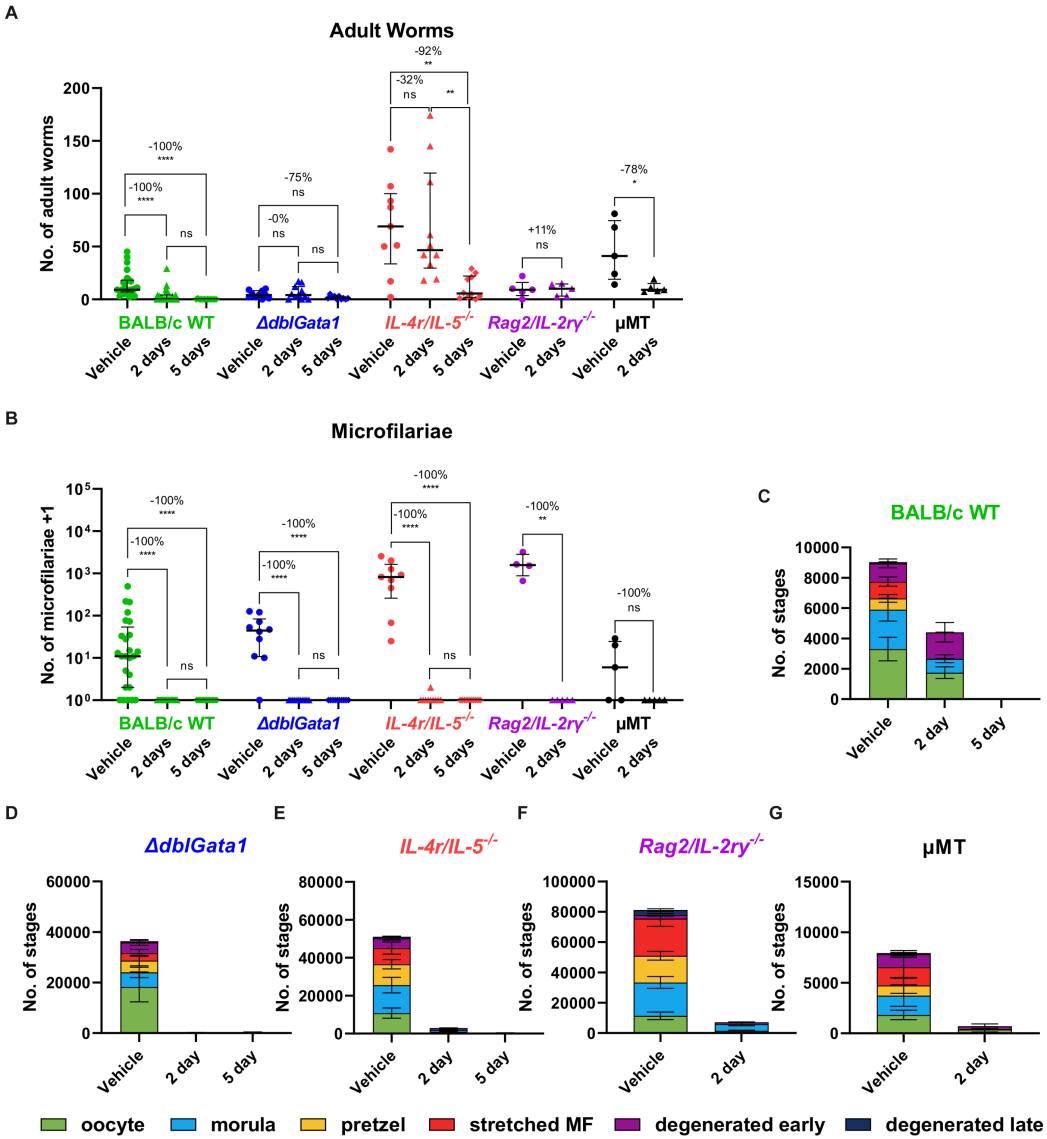
Reduced macrofilaricidal efficacy of flubendazole in immunodeficient mice. **(A–G)** Indicated mouse strains were naturally infected with *Litomosoides sigmodontis* and treated subcutaneously with 2 mg/kg flubendazole once per day for 2 or 5 days starting 35 days after the infection. Necropsies were performed 70 days after the infection. **(A)** Adult worm burden. **(B)** Microfilariae per 50 μL peripheral blood +1. **(C–G)** Average number of embryonal stages per female worm in **(C)** BALB/c (green), **(D)** *ΔdblGata1* (blue), **(E)** *IL-4r/IL-5^−/−^* (red), **(F)** *Rag2/IL-2rγ^−/−^* (purple) and **(G)** μMT mice (black). **(A,B)** Data shown as median with interquartile range. Numbers show reduction of median in comparison to corresponding vehicle control. **(C–G)** Data shown as mean ± SEM. **(A–G)** Data for BALB/c pooled from 5 experiments, *ΔdblGata1* and *IL-4r/IL-5^−/−^* pooled from 2 experiments, *Rag2/IL-2rγ^−/−^* and μMT from 1 experiment. Statistical analysis using Kruskal-Wallis with Dunn’s *post-hoc* test, *p < 0.05, **p < 0.01, ****p < 0.0001.

Similar to OXF, the macrofilaricidal efficacy of FBZ was reduced in all immunodeficient strains (Figure 2A). Treatment with FBZ in BALB/c WT mice led to a median reduction of 100% with 56 or 93% of animals cleared of all adult worms after 2 and 5 days of treatment, respectively (Table 2). By contrast, reductions in the immunodeficient strains ranged from 0 to 92% with significant reductions only in μMT mice after 2 days of FBZ and *IL-4r/IL-5^−/−^* mice after 5 days of FBZ treatment (Figure 2A). In addition, FBZ treatment only achieved a partial clearance of adult worms in the immunodeficient strains with the most significant effect in *ΔdblGata1* mice with 27 and 30% of animals being adult worm free, respectively (Table 2). Interestingly, the effect of FBZ on microfilariae was drastically different compared to the effect of OXF (Figure 2B). All mice with the exception of one *IL-4r/IL-5^−/−^* mouse had no microfilariae detectable in the peripheral blood after FBZ treatment (Table 2). Results from the analysis of the embryonal development supported the microfilariae data showing significant reductions in all embryonal stages after treatment (Figures 2C–G). Furthermore, late stages were almost completely eliminated in all strains, and only early or degenerated stages could be detected (Supplementary Table S3). Hence we conclude that the macrofilaricidal efficacy of FBZ is dependent on the immune system. However, the prevention of microfilaremia is immune system-independent.

**Table 2:** Clearance of adult worms and microfilariae after FBZ treatment.

	Adult worm negative mice			Microfilariae negative mice		
	Vehicle	2 days FBZ	5 days FBZ	Vehicle	2 days FBZ	5 days FBZ
BALB/c	0.0%	56.0%	93.3%	20.0%	100%	100%
	(0/25)	(14/25)	(14/15)	(5/25)	(25/25)	(15/15)
*ΔdblGata1*	10.0%	27.2%	30.0%	10.0%	100%	100%
	(1/10)	(3/11)	(3/10)	(1/10)	(11/11)	(10/10)
*IL-4r/* *IL-5^−/−^*	0.0%	0.0%	10.0%	0.0%	90%	100%
	(0/9)	(0/10)	(1/10)	(0/9)	(9/10)	(10/10)
*Rag2/* *IL-2rγ^−/−^*	0.0%	0.0%	n/a	0.0%	100%	n/a
	(0/5)	(0/5)		(0/5)	(5/5)	
μMT	0.0%	0.0%	n/a	20.0%	100%	n/a
	(0/5)	(0/5)		(2/5)	(5/5)	

BALB/c WT mice and immunodeficient strains (*ΔdblGata1*, *IL-4r/IL-5^−/−^,* μMT and *Rag2/IL-2rγ^−/−^*) were naturally infected with *Litomosoides sigmodontis* and treated with 2 mg/kg FBZ once per day for 2 or 5 days starting 35 dpi. Number of adult worms in the thoracic cavity and number of microfilariae per 50 μL blood were quantified 70 dpi. Data is pooled from 1 to 5 experiments. Shown is the number and frequency of animals that were free of adult worms and microfilariae at 70 dpi.

### Distinct patterns of immunological changes in different strains after benzimidazole treatment

**Figure 3:**
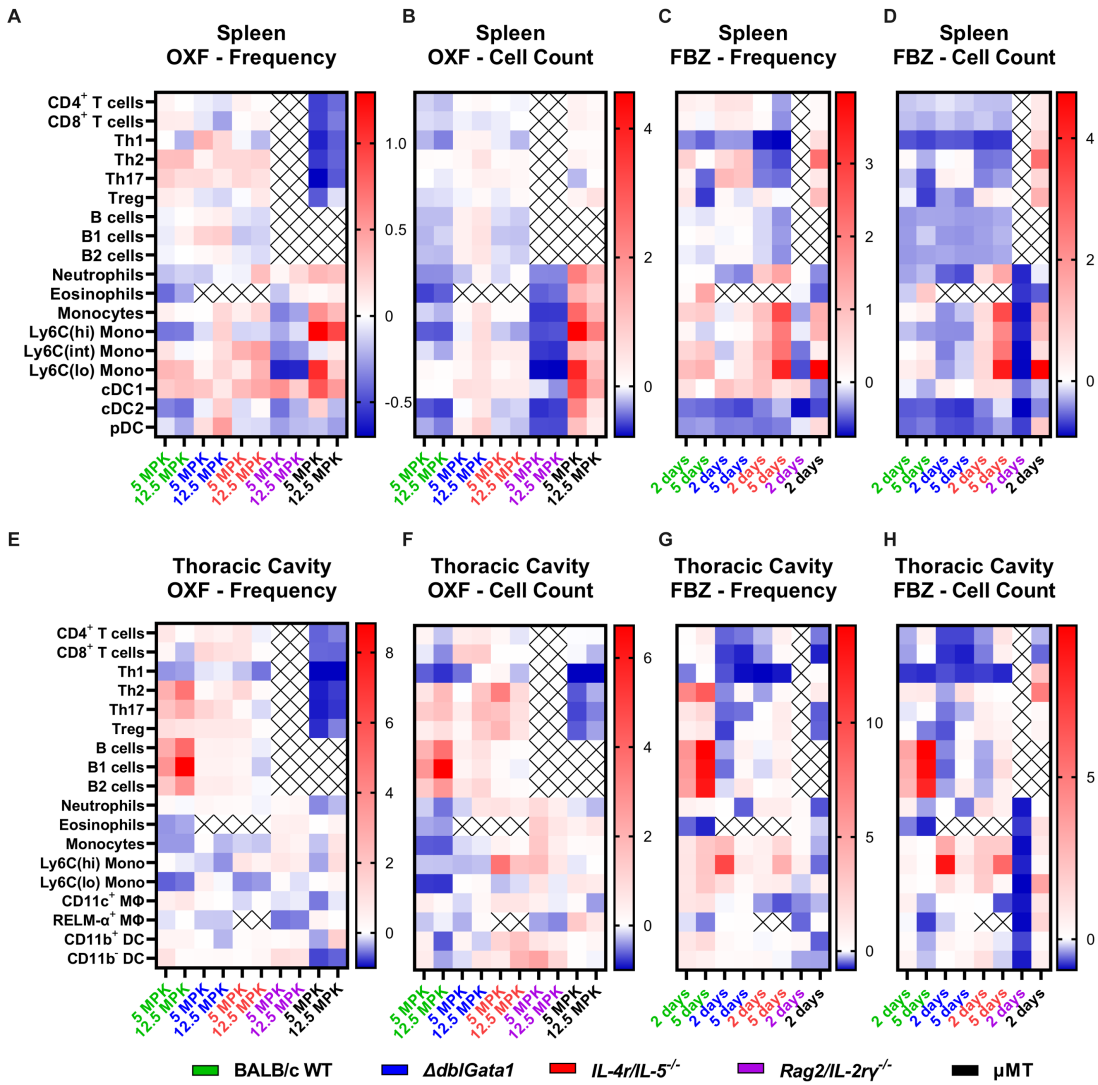
Distinct immunological changes in different immunodeficient strains after anti-filarial treatment. **(A–H)** Indicated mouse strains were naturally infected with *Litomosoides sigmodontis* and treated with **(A,B,E,F)** oxfendazole or **(C,D,G,H)** flubendazole 35 days after the infection. Necropsies were performed 70 days after the infection and immune cell populations in **(A–D)** spleen and **(E–H)** thoracic cavity were analyzed via flow cytometry. Heat maps show fold change of **(A,C,E,G)** mean of immune cell frequencies and **(B,D,F,H)** mean of total cell counts after treatment in comparison to corresponding vehicle controls. **(A,B,E,F)** Data for BALB/c (green) pooled from 6 experiments, *IL-4r/IL-5^−/−^* (red) pooled from 3 experiments, *ΔdblGata1* (blue), *Rag2/IL-2rγ^−/−^* (purple) and μMT (black) pooled from 2 experiments. **(C,D,G,H)** Data for BALB/c pooled from 5 experiments, *ΔdblGata1* and *IL-4r/IL-5^−/−^* pooled from 2 experiments, *Rag2/IL-2rγ^−/−^* and μMT from 1 experiment.

Next, we aimed to further characterize the impact of the host’s immune status by analyzing changes in immune cell populations in the thoracic cavity, the site of adult worm residency, and the spleen, which plays a crucial role in the elimination of microfilariae, after treatment (see Supplementary Figures S1, S2 for gating strategies). OXF and FBZ treatment led to distinct, strain-and compound-specific changes in both spleen (Figures 3A–D) and thoracic cavity (Figures 3E–H) compared to corresponding vehicle controls.

In BALB/c WT mice (shown in green in Figure 3), treatment with both OXF and FBZ induced only limited changes in cell frequencies in the spleen, with the strongest reductions observed for eosinophils, Ly6C(hi) monocytes and cDC2s after OXF treatment (Figure 3A) and Th1 cells, cDCs2 and pDCs after FBZ treatment (Figure 3C) while the total cell count was reduced for almost all cell types (Figures 3B,D). By contrast, treatment with OXF led to an overall increase in cell frequencies and total cell counts in *ΔdblGata1* mice (blue, Figures 3A,B). Treatment with FBZ in *ΔdblGata1* mice, on the other hand, led to broadly similar changes as in BALB/c WT mice with decreases in Th1, cDC2, and pDC frequencies and overall decreased total cell counts (Figures 3C,D). For *IL-4r/IL-5^−/−^* mice, treatment with OXF led to no significant changes in cell frequencies or total counts (red, Figures 3A,B), whereas FBZ treatment reduced frequencies and total cell counts of lymphocytes (except Tregs) while myeloid cells except for cDC2s and pDCs were increased (Figures 3C,D). *Rag2/IL-2ry^−/−^* had overall decreased frequencies and total cell counts after both OXF and FBZ treatment (purple, Figures 3A–D). μMT mice had decreased lymphocyte frequencies after OXF treatment, but unchanged or slightly elevated cell counts (black, Figures 3A,B) and overall increased lymphocyte numbers after FBZ treatment (Figures 3C,D). Interestingly, myeloid cell numbers were mostly increased after OXF treatment, whereas FBZ treatment reduced neutrophils, eosinophils and cDC numbers, while other myeloid cells were increased in μMT mice (Figures 3C,D).

In the thoracic cavity of WT mice, treatment with both OXF and FBZ led to an increase in most lymphocyte numbers except for Th1 cells (green, Figures 3E–H). Myeloid cells, on the other hand, were mostly decreased after OXF treatment or essentially unchanged, except for strongly decreased eosinophils after FBZ treatment (Figures 3E–H). For both *ΔdblGata1* and *IL-4r/IL-5^−/−^* mice, OXF treatment had only minor effects on cell numbers in the thoracic cavity, whereas treatment with FBZ led to a significant decrease in CD4^+^ (especially Th1) and CD8^+^ T cell numbers (blue and red, Figures 3E–H). For *Rag2/IL-2rγ^−/−^*, OXF treatment induced only limited changes in thoracic cavity cell numbers while treatment with FBZ strongly reduced total cell numbers (purple, Figures 3E–H). By contrast, treatment in μMT mice revealed more extensive changes after OXF rather than FBZ treatment (black, Figures 3E–H). Overall, OXF and FBZ induced marked differences in both spleen and thoracic cavity cell compositions with distinct differential patterns in each strain.

### Combination of oxfendazole with interleukin-5 improves macrofilaricidal efficacy against *Litomosoides sigmodontis*

**Figure 4:**
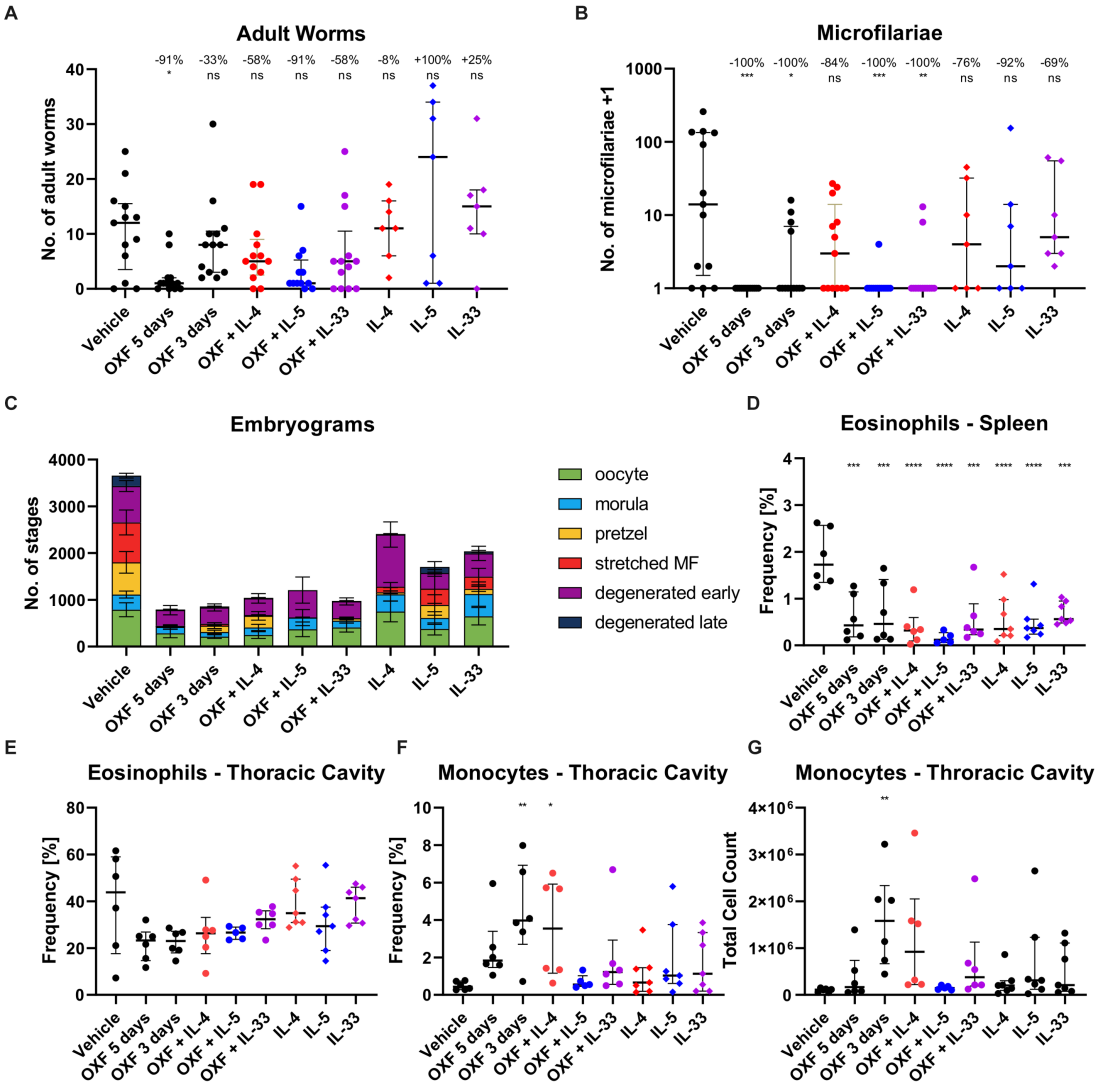
Combination of oxfendazole with interleukin-5 improves macrofilaricidal treatment efficacy in shortened treatment regimen. **(A–G)** Six-week old female BALB/c mice were naturally infected with *Litomosoides sigmodontis* and treated with 12.5 mg/kg oxfendazole twice per day for 5 days (positive control) or 3 days (shortened treatment) with or without addition of intranasal application of 2 μg IL-4, IL-5, or IL-33 once per day. Necropsies were performed 70 days after the infection. **(A)** Adult worm burden. **(B)** Microfilariae per 50 μL peripheral blood +1. **(C)** Average number of embryonal stages per female worm. **(D)** Frequency of eosinophils [CD8^−^, CD11b^+^, Ly6G^−^, Siglec-F^+^] in spleen. **(E)** Frequency of eosinophils [CD8^−^, CD11b^+^, Siglec-F^+^] in thoracic cavity. **(F)** Frequency of monocytes [CD8^−^, CD11b^+^, Siglec-F^−^, RELMα^−^, Ly6G^−^, I-ab^(lo)^] in thoracic cavity. **(G)** Total cell count of monocytes in thoracic cavity. **(A,B,D–G)** Data shown as median with interquartile range. Numbers show reduction of median in comparison to vehicle control. **(C)** Data shown as mean ± SEM. **(A–C)** Data for IL-4, IL-5, IL-33 from 1 experiment, data for other groups pooled from 2 experiments (*n* = 6–7 per group per experiment). **(D–G)** Representative data for two experiments. **(A-C,E)** Statistical analysis using Kruskal-Wallis with Dunn’s *post-hoc* test, **(D,F,G)** Statistical analysis using One-Way ANOVA with Dunnett’s multiple comparisons test, *p < 0.05, **p < 0.01, ***p < 0.001, ****p < 0.0001.

Based on the reduced treatment efficacy of OXF and FBZ observed in immunodeficient mice, we hypothesized that stimulating the immune system during drug treatment might improve the efficacy or enable an equally effective treatment using a shorter treatment regimen. To investigate this, we naturally infected BALB/c WT mice with *L. sigmodontis* and treated them with 12.5 mg/kg OXF for 3 days (suboptimal time) with or without additional intranasal application of IL-4, IL-5, or IL-33 starting 35 dpi (Figure 4). The cytokines were given intranasally to induce a local immune response at the site of infection, i.e., the thoracic cavity (Jackson-Jones et al., 2016).

Treatment with OXF for 3 days alone led to no statistically significant difference of the adult worm burden, with a 33% median reduction compared to the vehicle controls (Figure 4A). Addition of IL-4 and IL-33 improved the median adult worm burden reduction to 58%. The combination with IL-5 boosted the efficacy to 91% achieving a similar reduction as a 5-day OXF treatment even though all three combination therapies did not lead to statistically significant differences in comparison to the vehicle control (Dunn’s *post-hoc* test). However, the combination with IL-5 did lead to significant differences compared to both the vehicle control (*p* = 0.025) as well as the 3-day OXF treatment (*p* = 0.004) via direct comparison (Mann–Whitney U test). Treatment with the cytokines alone did not lead to a reduction of the adult worm burden (Figure 4A). Besides the improved macrofilaricidal efficacy, the combination of OXF with IL-5 reduced the number of microfilariae-positive animals from 4/13 to 1/13 compared to OXF alone (Figure 4B). Analysis of the embryonal development showed a substantial reduction of all stages after OXF treatment (Figure 4C). Importantly, significant numbers of late stages remained after 3-day OXF treatment (137.1 ± 61.8 pretzel stages, 36.7 ± 24.9 stretched microfilariae, mean ± SEM) which were almost completely eliminated in the OXF + IL-5 group (5.8 ± 4.3 pretzel stages, 0.0 ± 0.0 stretched microfilariae) (Supplementary Table S4). Based on these results, we also tested a treatment regimen of only 2 days. However, a combination therapy of 12.5 or 25 mg/kg OXF with IL-5 for only 2 days failed to significantly reduce the adult worm burden or microfilariae numbers (Supplementary Figure S3).

**Figure 5:**
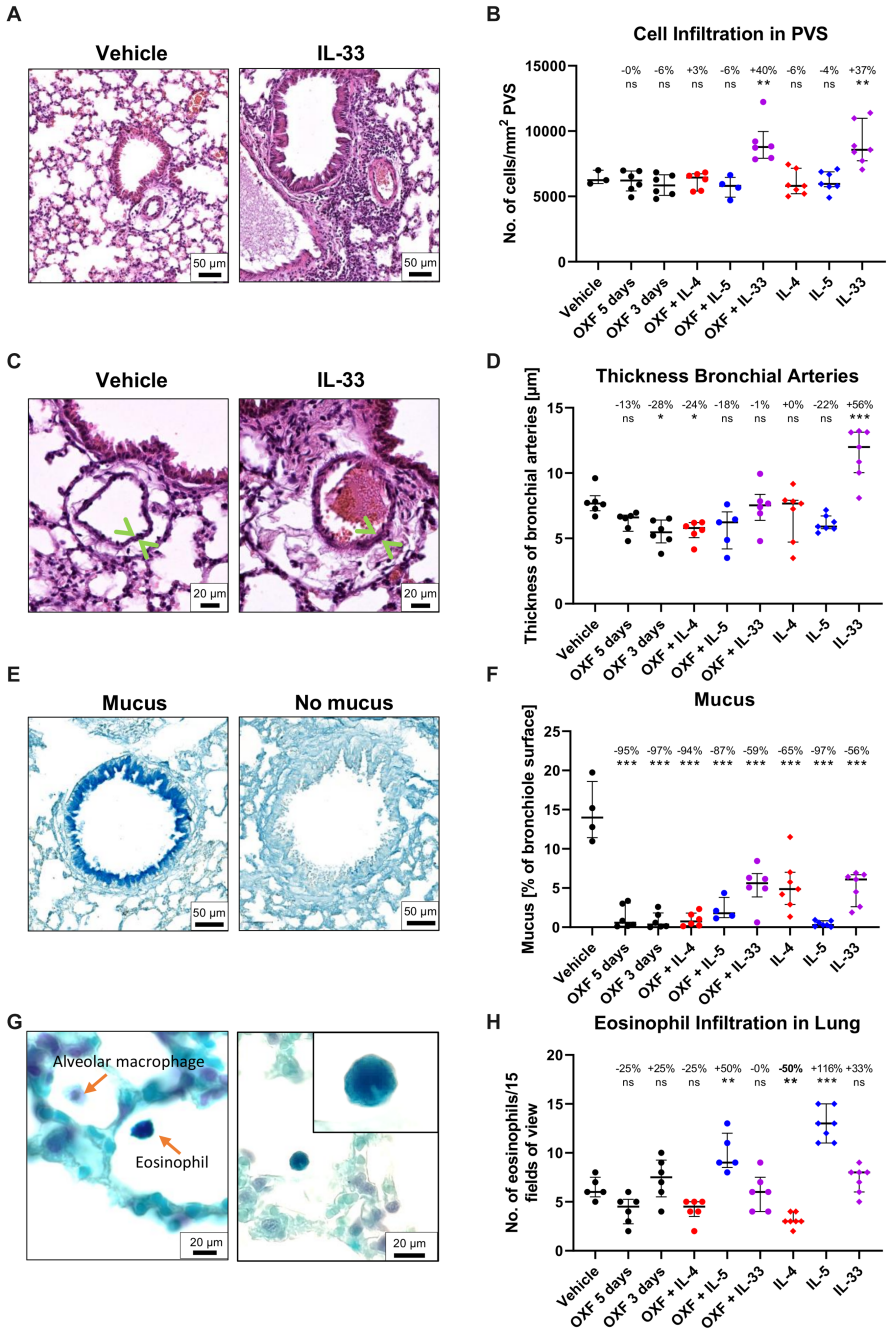
Histological changes in the lung after combination therapy. **(A–H)** Six-week old female BALB/c mice were naturally infected with *Litomosoides sigmodontis* and treated with 12.5 mg/kg oxfendazole twice per day for 5 days (positive control) or 3 days (shortened treatment) with or without addition of intranasal application of 2 μg IL-4, IL-5, or IL-33 once per day. Necropsies were performed 70 days after the infection and lungs were processed for histological analysis. **(A)** H&E staining of perivascular spaces (PVS). **(B)** Quantification of Hematoxylin positive nuclei per mm^2^ in the PVS. **(C)** H&E staining of bronchial arteries. Arrows indicate the artery boundaries. **(D)** Quantification of bronchial vein thickness. **(E)** Alcian blue staining of mucus in bronchial epithelium. **(F)** Quantification of mucus on bronchiole surface. **(G)** Luxol fast blue staining of eosinophils. Left image displays a Luxol blue positive eosinophil and an alveolar macrophage. The corner zoom on the right image highlights the eosinophil lobed nucleus. **(H)** Quantification of eosinophil infiltration in the lung. **(A–H)** Results are expressed as median with interquartile of *n* = 3–7 mice per group (1 experiment). Numbers show reduction of median in comparison to vehicle control. Statistical analysis using One-way ANOVA followed by Dunnett’s Multiple Comparison Tests, *p < 0.05, **p < 0.01, ***p < 0.001.

Interestingly, flow cytometric analysis of the 3-day OXF combination therapy revealed a reduction in eosinophil frequencies in the spleen in mice treated with OXF, cytokines (IL-4, IL-5, IL-33) or a combination thereof compared to the vehicle controls (Figure 4D). The composition of immune cells in the thoracic cavity was mostly unchanged after treatment with an intriguing increase in monocyte numbers after 3-day OXF treatment (Figures 4F,G). To assess any pathological changes in adjacent organs caused by the combination therapy, we analyzed histological changes in the lungs of treated mice (Figure 5). Here, we observed that the treatment with IL-33 appeared to cause vascular inflammation with increased cell infiltration into the pulmonary vascular stroma (Figures 5A,B) and increased thickness of bronchial arteries (Figures 5C,D). Apart from these pathological changes, treatment with OXF, the combination of OXF with cytokines and the cytokines by themselves led to a reduction in mucus covering the surfaces of bronchioles (Figures 5E,F). Lastly, as expected, treatment with IL-5 led to an infiltration of eosinophils into the lung (Figures 5G,H).

Overall, the combination of OXF with IL-5 improved the macrofilaricidal efficacy, reduced the number of microfilariae-positive animals, and no pathological changes were observed in the lungs of treated mice.

## Discussion

In the present study, we characterized the role of the immune system during anti-filarial treatment in the *L. sigmodontis* rodent model and investigated the potential of a combination therapy approach to improve the macrofilaricidal efficacy of OXF.

In the first part, we compared the treatment efficacy and immunological changes after treatment with two different benzimidazole compounds, OXF and FBZ, in BALB/c WT mice with immunodeficient mouse strains, i.e., *ΔdblGata1* mice lacking eosinophils, *IL-4r/IL-5^−/−^* mice lacking eosinophils as well as RELMα^+^ and mature F4/80 (high) macrophages, μMT mice lacking mature B cells and antibodies and *Rag2/IL-2rγ^−/−^* mice lacking T cells, B cells, NK cells and ILCs. The macrofilaricidal efficacy of both OXF and FBZ was reduced in all tested immunodeficient strains, and treatment efficacy was lowest in strains with more severe immunodeficiency, i.e., *IL-4r/IL-5^−/−^* vs. *ΔdblGata1* mice, or completely abrogated in *Rag2/IL-2rγ^−/−^* mice. Interestingly, the effect on microfilariae, the filarial progeny, which are released into the peripheral blood, were markedly different for both drugs 70 dpi. While treatment with FBZ led to a complete absence of microfilariae in all strains 70 dpi, treatment with OXF prevented microfilaremia only in BALB/c WT and μMT mice 70 dpi, indicating that the effect of OXF on microfilaremia is dependent on the immune system overall but independent of mature B cells and antibodies. At 56 dpi, mice treated with either OXF or FBZ presented with 0 microfilariae in the peripheral blood except for two *ΔdblGata1* mice (Supplementary Figure S4). Analysis of embryonal development supported these results with a near complete absence of late stages after FBZ treatment in all strains or only BALB/c and μMT mice after OXF treatment.

Notably, treatment with FBZ and OXF was performed between 35 and 39 dpi, i.e., after the final molt into adult worms (~30 dpi) but before the development of microfilariae (~50 dpi) (Petit et al., 1992; Hübner et al., 2009; Risch et al., 2021). In addition, FBZ was injected subcutaneously while OXF was given orally. Prior studies in jirds (*Meriones unguiculatus*) have shown that subcutaneously injected FBZ is slowly released, and FBZ remains stable and detectable in the plasma for >50 days after injection (Hübner et al., 2019). Orally given OXF, on the other hand, is rapidly metabolized with a T_1/2_ of 2.8 h in mice (Hübner et al., 2020). Therefore, OXF only interacted with the adult worms and prevented microfilaremia purely via an impact on the fertility of adult worms, whereas FBZ may have affected both adult worms and microfilariae. It is, however, relevant to note that both OXF and FBZ have been reported to be relatively ineffective against the microfilarial stage of *L. sigmodontis* and *B. malayi,* respectively. Prior studies have posited the damage to adult worms and subsequent negative impact on fertility as the leading cause for the reduced microfilariae burden for both parasites (Hübner et al., 2019; Sjoberg et al., 2019).

Previous studies have reported that *ΔdblGata1* mice present with a higher susceptibility to *L. sigmodontis* infection than BALB/c WT mice with an increased adult worm burden and > 70% of mice developing microfilaremia (Fercoq et al., 2019; Frohberger et al., 2019). In the current study, we have observed a significantly lower number of microfilariae-positive animals only in the controls for the OXF treatment but not the FBZ treatment (Figures 1B, 2B). A more detailed analysis of the data sets revealed that this discrepancy is due to a strong sexual dimorphism in *ΔdblGata1* mice, with female mice significantly more susceptible to develop patent infections (Supplementary Figure S5).

The flow cytometry data analysis revealed significant changes after treatment in all strains with distinct strain-specific patterns in both the spleen and thoracic cavity. The thoracic cavity, which contains the adult worms of *L. sigmodontis,* is filled with a variety of immunologically active components such as lysozymes, antibodies, complement factors and different immune cells depending on the stage of infection (Miserocchi, 1997; Charalampidis et al., 2015; Finlay and Allen, 2020). Of note, eosinophils are absent from the thoracic cavity in naïve mice but recruited during the infection starting from day 11 after the infection (Finlay and Allen, 2020; Ehrens et al., 2022b). Flow cytometry analysis showed decreased eosinophil frequencies and total cell counts after OXF treatment in the thoracic cavity in BALB/c but an increase in *Rag2/IL-2rγ^−/−^* and μMT mice. As eosinophils are critical effectors in controlling the adult worm burden (Ehrens et al., 2022b), it is possible that this strain-dependent difference can be explained by the clearance of adult worms in BALB/c mice and the continuous presence of the adult worms in the immunodeficient strains. Similarly, BALB/c mice presented with markedly increased B and Th2 cell numbers which were either absent or less pronounced in the other strains. Both T and B cells play important roles in worm killing and mediating protection against secondary infections (Al-Qaoud et al., 1997, 1998; Martin et al., 2001; Finlay and Allen, 2020). Overall, the data set highlights several intriguing immunological changes after treatment in both immunocompetent WT and immunodeficient KO mice.

Based on the results from the immunodeficient mice, we next investigated the potential of a combination therapy of OXF with three cytokines (IL-4, IL-5, and IL-33). The cytokines were chosen based on the reduced treatment efficacy in *ΔdblGata1* and *IL-4r/IL-5^−/−^* mice and to boost the type 2 immune response typically associated with helminth infections (Finlay and Allen, 2020; Ehrens et al., 2022b). The combination with IL-5 achieved the most promising results with improved macrofilaricidal efficacy, reduction in microfilariae-positive animals and no pathological changes in the lungs of treated mice. The combination of anthelmintics with immunostimulatory compounds has already been tested for the treatment of several different helminths including *Angiostrongylus cantonensis* (mebendazole with IL-12), *B. malayi* (diethylcarbamazine with tuftsin, mebendazole with Freund’s complete adjuvant), *Echinococcus granulosus* (albendazole with IL-12, IFN-α, and IFN-γ), *Echinococcus multilocularis* (albendazole with transfer factor), *L. sigmodontis* (ivermectin with various immunomodulators), *Schistosoma mansoni* (praziquantel with various immunomodulators) and *Toxocara canis* (albendazole with glucan); most studies reported an overall improvement of the treatment efficacy (Murthy et al., 1992; Fatma et al., 1994; Hrckova and Velebny, 2001; Du et al., 2003; Owais et al., 2003; Dvoroznakova et al., 2009; Zhang et al., 2017; Rahdar et al., 2020; Silva et al., 2020). For example, Zhang et al. demonstrated a significantly stronger reduction of the number, size and weight of *E. granulosus* cysts in mice treated with albendazole + IFN-α compared to mice treated with albendazole alone. In addition, they reported structural modifications of cysts associated with the combination therapy (Zhang et al., 2017). Murthy et al. showed that mebendazole given intraperitoneally, along with Freund’s complete adjuvant, was four times more effective as a filaricide than mebendazole alone (Murthy et al., 1992). To the best of the authors’ knowledge, the current study is the first to investigate the potential of improving the anti-filarial activity of OXF via a combination with immunostimulatory compounds and may serve as a proof of concept study for further research in this area.

OXF has been used as a broad-spectrum anthelmintic in the veterinary market since the 1990s (Gonzalez et al., 2019). However, concerted efforts by academia and the Drugs for Neglected Disease initiative (DNDi) have investigated the potential to repurpose OXF as a pan-nematode compound for use in humans [Specht and Keiser, 2022; Ehrens et al., 2022a, Helminth Elimination Platform (HELP)]^3^. We have previously shown the activity of OXF against *L. sigmodontis* adult worms, *O. gutturosa* adult worms and *O. volvulus* pre-adult stages (Hübner et al., 2020). In addition, first tolerability and safety studies in humans have shown OXF to be well tolerated at relevant dosages (Bach et al., 2020). OXF is currently transitioning into phase II clinical trials and will be evaluated as a potential pan-nematode drug for *O. volvulus*, *L. loa*, *M. perstans*, and *Trichuris trichiura* in the Democratic Republic of the Congo, Gabon, Cameroon and Tanzania as part of the recently launched EU-EDCTP3 project eWHORM (enabling the WHO Road Map, see text footnote 2).

In light of this, further research into a combination of OXF with immunostimulatory compounds may yield additional treatment options. However, several open questions remain. One particular area that requires further research is the choice of immunostimulant. The cytokines utilized in this proof of concept study (IL-4, IL-5, and IL-33) are not likely to be applicable in the human setting as (1) recombinant cytokines are too expensive for anti-filarial treatment in the affected regions and (2) recombinant cytokines would require a lengthy regulatory approval. On the other hand, the combination of OXF with already approved immunostimulatory compounds/treatments such as pidotimod (used mainly for respiratory diseases) or commonly used vaccine adjuvants may allow a more cost-effective treatment, shorter treatment regimens and lower drug concentrations and a faster translation of basic research to human patients and therefore contribute to the critical actions outlined in the WHO NTD roadmap 2021–2030 (Mahashur et al., 2019; WHO, 2020). One other yet unanswered question is the role of the immune system during anti-*Wolbachia* treatment.

Overall, in this study, we have demonstrated the significant contributions of the immune system during anti-filarial treatment. Using various immunodeficient mouse strains, we have highlighted how the absence of different immune cells or components affects treatment efficacy against adult worms, microfilariae and embryogenesis of *L. sigmodontis*. In addition, we have shown how the combination of OXF with immunostimulatory compounds can improve treatment efficacy, and further research may yield more treatment options for human filarial patients.

## Data availability statement

The original contributions presented in the study are included in the article/Supplementary material, further inquiries can be directed to the corresponding author.

## Ethics statement

The animal study was reviewed and approved by the Landesamt für Natur-, Umwelt-und Verbraucherschutz, AZ: 84–02.04.2015.A507, 81–02.04.2020.A244, 81–02.05.40.18.057.

## Author contributions

FR, CM, AH, and MH: conceptualization. FR, JG, FF, and MH: data curation. FR, JS, JR, BL, AE, JG, FF, MK, MF, and CM: experimentation. CM, AH, and MH: resources. CM and MH: supervision. FR and MH: writing—original draft. All authors: writing—review and editing. All authors contributed to the article and approved the submitted version.

## Funding

JR and JS were supported by a PhD scholarship from the Jürgen Manchot Stiftung, Düsseldorf, Germany. AH was funded by the Deutsche Forschungsgemeinschaft (DFG, German Research Foundation) under Germany’s Excellence Strategy EXC 1023. AH and MH were funded under Germany’s Excellence Strategy—EXC2151-390873048. AH and MH were members of the German Center for Infection Research (DZIF). MH received funding from the German Center for Infection Research (TTU 09.701).

## Conflict of interest

The authors declare that the research was conducted in the absence of any commercial or financial relationships that could be construed as a potential conflict of interest.

## Publisher’s note

All claims expressed in this article are solely those of the authors and do not necessarily represent those of their affiliated organizations, or those of the publisher, the editors and the reviewers. Any product that may be evaluated in this article, or claim that may be made by its manufacturer, is not guaranteed or endorsed by the publisher.

## Footnotes

^1^ https://eliminateworms.org/

^2^ https://www.ewhorm.org/

^3^ https://dndi.org/research-development/portfolio/oxfendazole/
