# Supplementary material for: The efficacy of the benzimidazoles oxfendazole and flubendazole against Litomosoides sigmodontis is dependent on the adaptive and innate immune system
Source: Front Microbiol. 2023 Jun 27;14:1213143. doi: 10.3389/fmicb.2023.1213143 (PMC10335397; doi:10.3389/fmicb.2023.1213143)
Supplement: Supplementary file 1 [file Data_Sheet_1.zip › Supplementary Figures S1-S5.pdf]

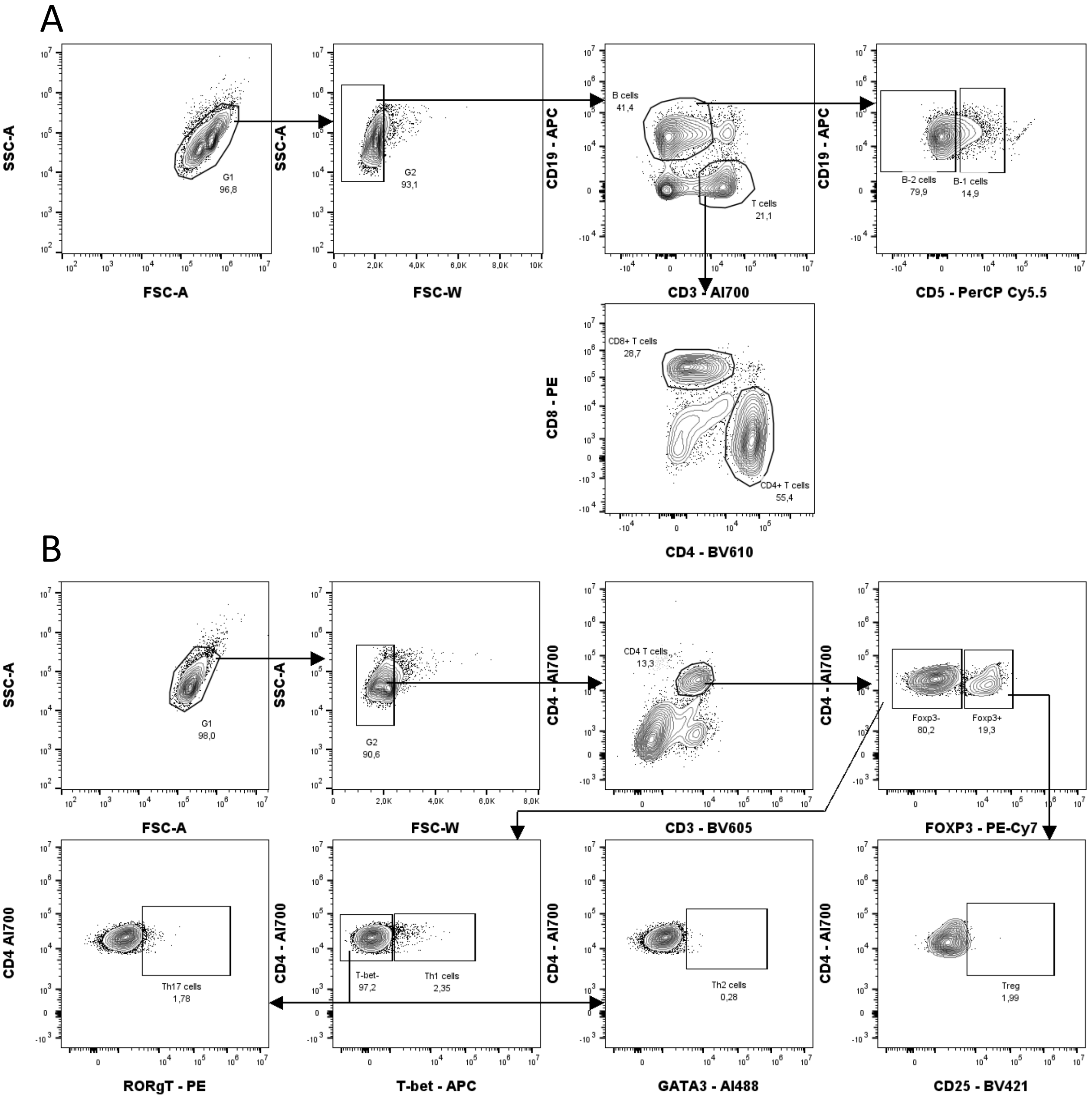

Supp. Fig. 1: Gating strategy to identify lymphocytes. Representative flow cytometry plots for the identification of lymphocytes **(A)** and T helper subsets **(B)**.

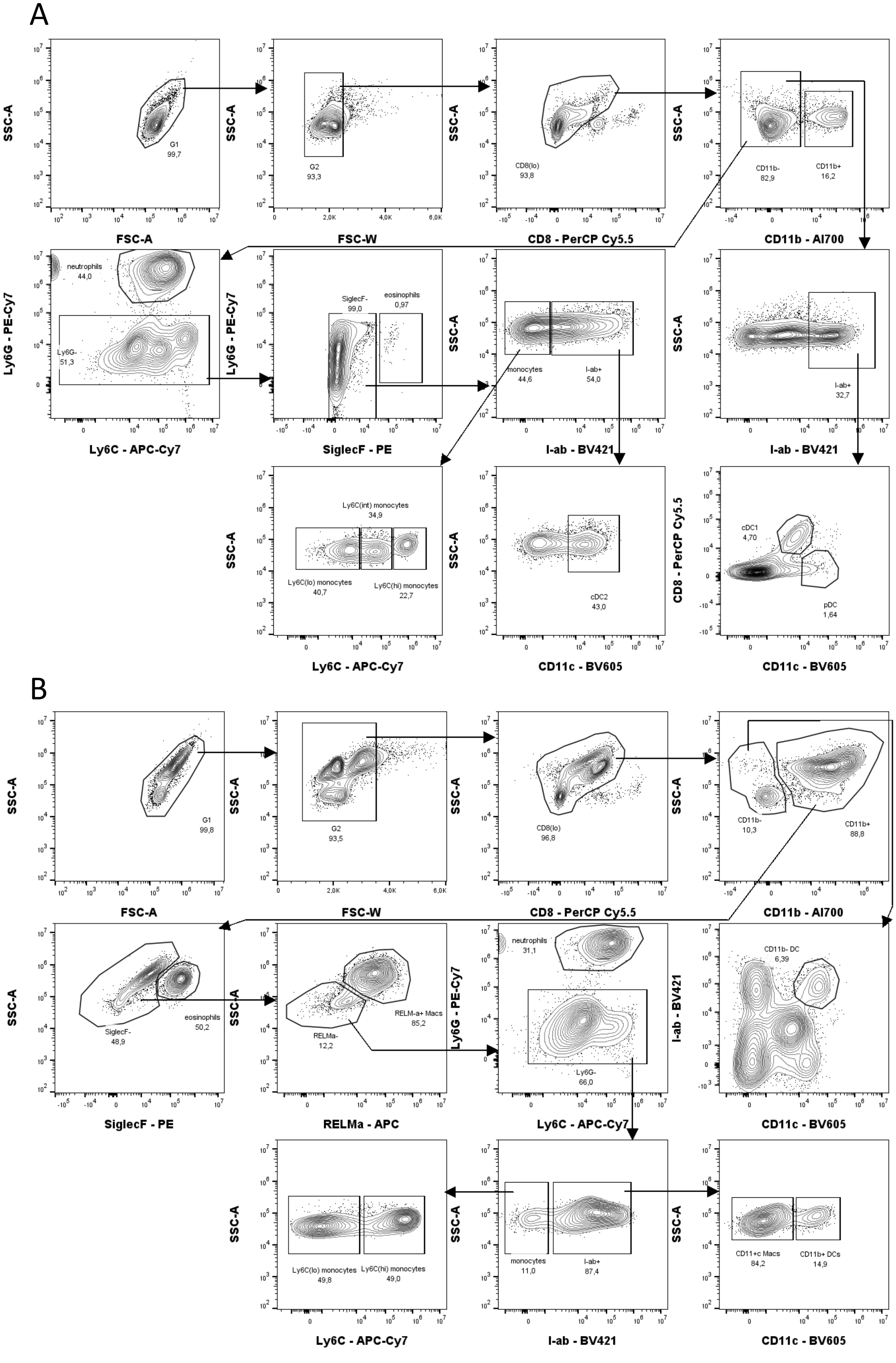

Supp. Fig. 2: Gating strategy to identify myeloid cells. Representative flow cytometry plots for the identification of myeloid cells in the spleen **(A)** and thoracic cavity **(B)**.

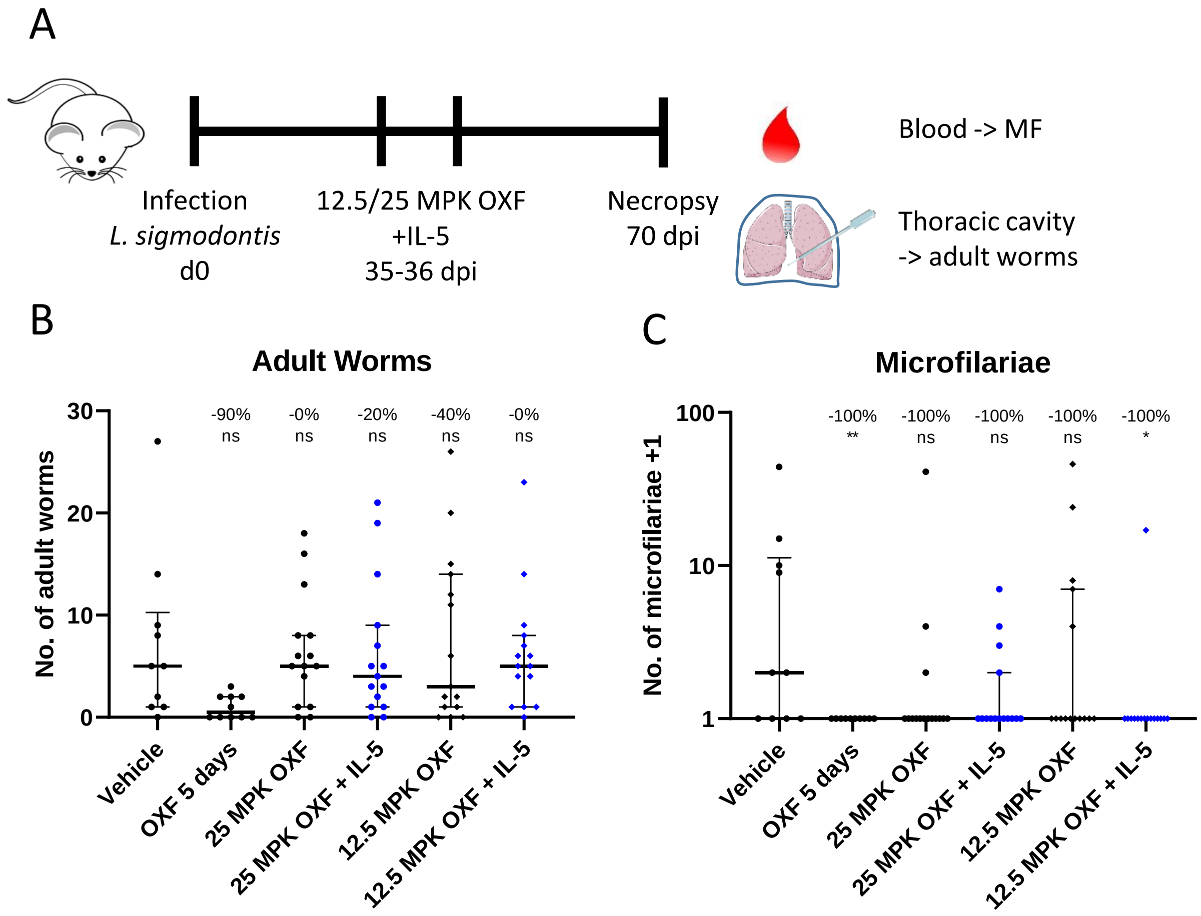

Supp. Fig. 3: Two-day combination therapy has no macrofilaricidal effect against *Litomosoides sigmodontis*. (A-C) 6-week-old female BALB/c mice were naturally infected with *Litomosoides sigmodontis* and treated with 12.5 or 25 mg/kg oxfendazole twice per day for 5 days (positive control) or 2 days (shortened treatment) with or without addition of intranasal application of 2 µg IL-5 once per day. Necropsies were performed 70 days after the infection. **(A)** Experimental setup. **(B)** Adult worm burden. **(C)** Microfilariae per 50 µl peripheral blood + 1. **(B-C)** Data shown as median with interquartile range. Numbers show reduction of median in comparison to vehicle control. Statistical analysis using Kruskal-Wallis with Dunn's post hoc test, * $p<0.05$, ** $p<0.01$.

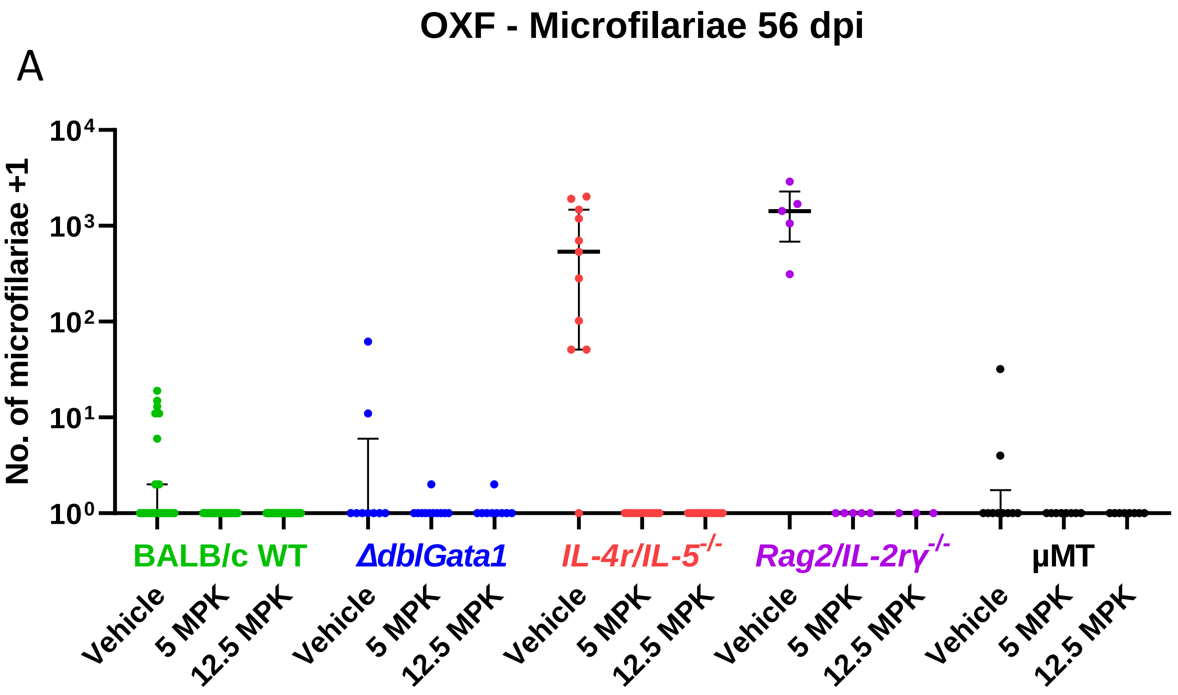

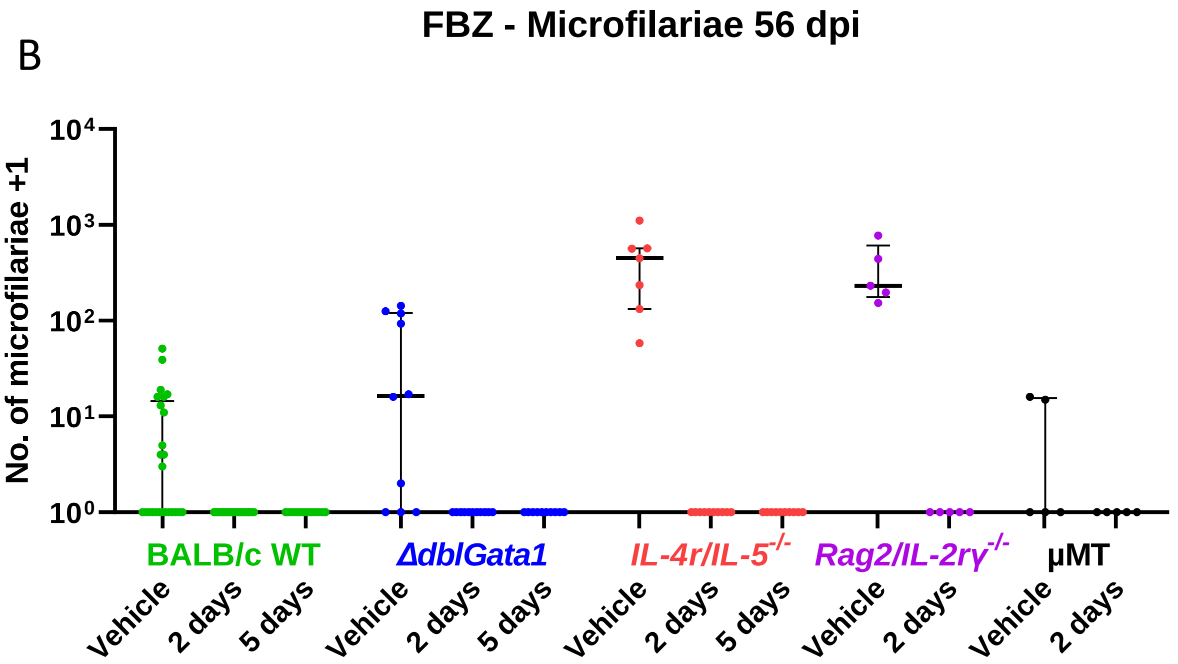

Supp. Fig. 4: Delay of microfilaremia after benzimidazole treatment. Indicated mouse strains were naturally infected with *Litomosoides sigmodontis* and treated **(A)** orally with 5 or 12.5 mg/kg oxfendazole twice per day for 5 days starting 35 days or **(B)** subcutaneously with 2 mg/kg flubendazole once per day for 2 or 5 days starting 35 days after the infection. 50 µl of blood was taken from the facial vein to quantify MF numbers in the peripheral blood 56 dpi. **(A)** Microfilariae per 50 µl peripheral blood + 1 for mice treated with OXF. **(B)** Microfilariae per 50 µl peripheral blood + 1 for mice treated with FBZ. **(A-B)** Data shown as median with interquartile range. **(A)** Data for BALB/c pooled from 6 experiments, *IL-4r/IL-5*$^{-/-}$ pooled from 3 experiments, *ΔdblGata1*, *Rag2/IL-2rγ*$^{-/-}$ and µMT pooled from 2 experiments. **(B)** Data for BALB/c pooled from 5 experiments, *ΔdblGata1* and *IL-4r/IL-5*$^{-/-}$ pooled from 2 experiments, *Rag2/IL-2rγ*$^{-/-}$ and µMT from 1 experiment.

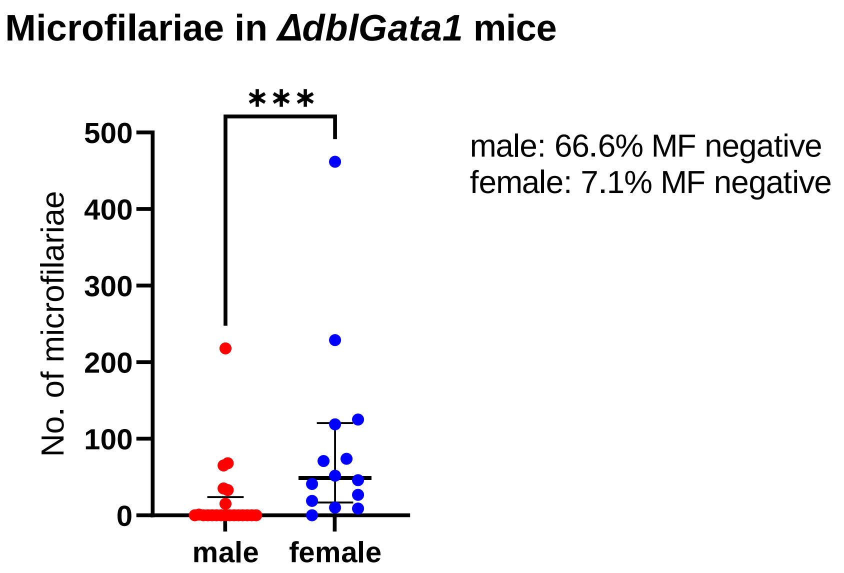

Supp. Fig. 5: Sexual dimorphism in susceptibility to microfilaremia. 6-20 week old female and male *ΔdblGata1* mice were naturally infected with *Litomosoides sigmodontis* and microfilariae numbers per 50 µl peripheral blood were determined 70 dpi. Data shown as median with interquartile range. Pooled data from 7 experiments (n = 21 male mice, 14 female mice). Statistical analysis using Mann-Whitney U test, *** p<0.001.
