# Supplementary material for: The efficacy of the benzimidazoles oxfendazole and flubendazole against Litomosoides sigmodontis is dependent on the adaptive and innate immune system
Source: Front Microbiol. 2023 Jun 27;14:1213143. doi: 10.3389/fmicb.2023.1213143 (PMC10335397; doi:10.3389/fmicb.2023.1213143)
Supplement: Supplementary file 1 [file Data_Sheet_1.zip › Supplementary Tables S2-S4.docx]

**Supp. Tab. 2: Effect of oxfendazole on embryonal development in immunodeficient mice**. Different mouse strains were naturally infected with *Litomosoides sigmodontis* and treated orally with 5 or 12.5 mg/kg oxfendazole twice per day for 5 days 35 days after the infection. Necropsies were performed 70 days after the infection. Embryonal development was analyzed in up to 5 intact female worms per mouse. Data for BALB/c pooled from 6 experiments, *IL-4r/IL-5^-/-^* pooled from 3 experiments, *ΔdblGata1*, *Rag2/IL-2rγ^-/-^* and µMT pooled from 2 experiments. Statistical analysis using Kruskal-Wallis with Dunn’s post-hoc test, * p<0.05, ** p<0.01, *** p<0.001, **** p<0.0001.

| Strain | Treatment | Oocyte | | Morula | | Pretzel | | Stretched MF | | Degenerated early | | Degenerated late | |
| --- | --- | --- | --- | --- | --- | --- | --- | --- | --- | --- | --- | --- | --- |
|  |  | **Mean ± SEM**  **stat. significance** | | **Mean ± SEM**  **stat. significance** | | **Mean ± SEM**  **stat. significance** | | **Mean ± SEM**  **stat. significance** | | **Mean ± SEM**  **stat. significance** | | **Mean ± SEM**  **stat. significance** | |
| BALB/c | **Vehicle** | 2019.4 ± 303.7 |  | 2259.9  ± 417.1 |  | 216.9  ± 78.6 |  | 484.8  ± 225.7 |  | 1390.9  ± 355.8 |  | 206.1  ± 136.7 |  |
|  | **5 MPK** | 853.6  ± 249.7 | ** | 540.7  ± 205.4 | ** | 111.0  ± 54.8 | ns | 15.5  ± 9.6 | ns | 334.2  ± 161.2 | **** | 46.2  ± 25.6 | ns |
|  | **12.5 MPK** | 1281.2 ± 312.7 | * | 1216.1  ± 450.9 | ns | 7.5  ± 4.4 | ns | 0.3  ± 0.3 | * | 453.9  ± 126.4 | * | 1.5  ± 1.2 | ns |
| *ΔdblGata1* | **Vehicle** | 1847.5 ± 345.0 |  | 1829.2  ± 762.9 |  | 427.7  ± 257.6 |  | 282.2  ± 216.6 |  | 531.6  ± 96.2 |  | 68.8  ± 39.7 |  |
|  | **5 MPK** | 1177.9 ± 564.2 | ** | 678.7  ± 259.3 | ns | 67.0  ± 28.3 | ns | 15.0  ± 8.5 | ns | 722.5  ± 221.1 | ns | 12.9  ± 8.4 | ns |
|  | **12.5 MPK** | 128.1  ± 37.2 | **** | 1079.1  ± 504.4 | ns | 157.2  ± 75.0 | ns | 65.5  ± 43.3 | ns | 570.9  ± 149.2 | ns | 19.0  ± 10.0 | ns |
| *IL-4r/*  *IL-5^-/-^* | **Vehicle** | 4885.1 ± 765.4 |  | 12104.0 ±1320.7 |  | 7961.7 ± 983.8 |  | 10902.0 ±2721.1 |  | 5561.7  ± 662.7 |  | 1772.3  ± 335.8 |  |
|  | **5 MPK** | 561.2  ± 149.4 | **** | 1295.5 ± 397.4 | **** | 667.4  ± 166.6 | **** | 41.7  ± 15.9 | **** | 823.4  ± 203.0 | **** | 68.7  ± 22.2 | **** |
|  | **12.5 MPK** | 322.1  ± 63.6 | **** | 580.2  ± 121.5 | **** | 469.4  ± 112.0 | **** | 19.2  ± 9.7 | **** | 1012.0  ± 192.4 | **** | 23.5  ± 7.4 | **** |
| µMT | **Vehicle** | 856.9  ± 159.2 |  | 616.1  ± 200.6 |  | 596.1  ± 287.7 |  | 850.0  ± 447.6 |  | 433.0  ± 85.0 |  | 173.0  ± 82.1 |  |
|  | **5 MPK** | 688.9  ± 252.6 | ns | 193.1  ± 109.3 | * | 1.5  ± 1.5 | * | 0.0  ± 0.0 | * | 260.5  ± 74.9 | ns | 0.0  ± 0.0 | * |
|  | **12.5 MPK** | 220.0 ± 125.0 | *** | 180.0 ± 133.61 | * | 0.0 ± 0.0 | * | 0.0 ± 0.0 | ns | 259.2 ± 106.8 | ns | 0.0 ± 0.0 | * |
| *Rag2/*  *IL-2rγ^-/-^* | **Vehicle** | 2018.8 ± 558.4 |  | 9287.5 ±1387.0 |  | 7537.5 ± 936.9 |  | 16256.0 ±2337.3 |  | 1250.0  ± 263.6 |  | 950.0  ± 237.0 |  |
|  | **5 MPK** | 473.3  ± 136.7 | ns | 573.3  ± 181.0 | *** | 540.0  ± 148.9 | **** | 226.6  ± 94.8 | **** | 720.0  ± 125.4 | ns | 53.3  ± 23.6 | * |
|  | **12.5 MPK** | 255.5  ± 64.7 | ns | 188.8  ± 65.4 | **** | 355.5  ± 170.8 | **** | 11.1  ± 11.1 | **** | 933.3  ± 180.2 | ns | 33.3  ± 33.3 | ** |

**Supp. Tab. 3: Effect of flubendazole on embryonal development in immunodeficient mice**. Different mouse strains were naturally infected with *Litomosoides sigmodontis* and treated subcutaneously with 2 mg flubendazole once per day for 2 or 5 days 35 days after the infection. Necropsies were performed 70 days after the infection. Embryonal development was analyzed in up to 5 intact female worms per mouse. Data for BALB/c pooled from 5 experiments, *ΔdblGata1* and *IL-4r/IL-5^-/-^* pooled from 2 experiments, *Rag2/IL-2rγ^-/-^* and µMT from 1 experiment. Statistical analysis using Kruskal-Wallis with Dunn’s post-hoc test, * p<0.05, ** p<0.01, *** p<0.001, **** p<0.0001.

| Strain | Treatment | Oocyte | | Morula | | Pretzel | | Stretched MF | | Degenerated early | | Degenerated late | |
| --- | --- | --- | --- | --- | --- | --- | --- | --- | --- | --- | --- | --- | --- |
|  |  | **Mean ± SEM**  **stat. significance** | | **Mean ± SEM**  **stat. significance** | | **Mean ± SEM**  **stat. significance** | | **Mean ± SEM**  **stat. significance** | | **Mean ± SEM**  **stat. significance** | | **Mean ± SEM**  **stat. significance** | |
| BALB/c | **Vehicle** | 3301.6 ± 779.5 |  | 2598.3 ± 754.8 |  | 740.5  ± 258.1 |  | 1108.9 ± 303.0 |  | 1201.7  ± 289.3 |  | 79.5  ± 25.2 |  |
|  | **2 days** | 1748.4 ± 380.8 | ns | 917.8  ± 258.3 | ns | 3.8  ± 3.8 | *** | 0.0  ± 0.0 | *** | 1737.7  ± 645.8 | ns | 0.3  ± 0.3 | * |
|  | **5 days** | No worms | | | | | | | | | | | |
| *ΔdblGata1* | **Vehicle** | 18276.0 ±5901.0 |  | 5741.2 ±2084.1 |  | 4688.2 ±2061.2 |  | 3023.5 ±1350.0 |  | 4123.5  ±1127.6 |  | 535.2  ± 332.8 |  |
|  | **2 days** | 46.4  ± 32.4 | **** | 0.6  ± 0.4 | **** | 0.0  ± 0.0 | **** | 0.0  ± 0.0 | **** | 131.5  ± 49.5 | **** | 5.2  ± 5.2 | ns |
|  | **5 days** | 59.8  ± 23.8 | *** | 0.2  ± 0.1 | **** | 0.0  ± 0.0 | **** | 0.0  ± 0.0 | *** | 272.7  ± 101.8 | ** | 0.0  ± 0.0 | ns |
| *IL-4r/*  *IL-5^-/-^* | **Vehicle** | 10800.0 ±2691.3 |  | 14770.0 ±4063.5 |  | 11000.0 ±2478.2 |  | 8555.6 ±3193.8 |  | 5148.1  ± 981.7 |  | 859.2  ± 227.6 |  |
|  | **2 days** | 1005.2 ± 205.4 | ** | 975.4  ± 716.3 | **** | 141.94 ± 141.9 | **** | 0.0 ± 0.0 | *** | 771.2  ± 147.2 | *** | 3.2  ± 3.2 | **** |
|  | **5 days** | 50.0 ± 45.4 | **** | 0.0 ± 0.0 | **** | 0.0 ± 0.0 | **** | 0.0 ± 0.0 | ** | 222.7 ± 108.3 | **** | 0.0 ± 0.0 | **** |
| µMT | **Vehicle** | 1820.0 ± 468.5 |  | 1913.3 ±1058.4 |  | 1006.7 ± 780.1 |  | 1820.0 ±1110.1 |  | 1286.7  ± 335.6 |  | 93.3  ± 57.2 |  |
|  | **2 days** | 389.2  ± 264.3 | **** | 37.1  ± 35.6 | *** | 0.0  ± 0.0 | ns | 0.0  ± 0.0 | ns | 270.7  ± 240.8 | ** | 0.0  ± 0.0 | ns |
|  | **5 days** | n/a (experiment not performed) | | | | | | | | | | | |
| *Rag2/*  *IL-2rγ^-/-^* | **Vehicle** | 11389.0 ±2474.6 |  | 22011.0 ±3870.0 |  | 17611.0 ± 2927.1 |  | 24456.0 ±5159.1 |  | 2511.1  ± 722.7 |  | 3155.6  ± 810.8 |  |
|  | **2 days** | 1623.5 ± 296.4 | **** | 4370.6 ±1153.7 | *** | 0.0  ± 0.0 | **** | 0.0  ± 0.0 | **** | 1135.3  ± 239.1 | ns | 0.0  ± 0.0 | **** |
|  | **5 days** | n/a (experiment not performed) | | | | | | | | | | | |

**Supp. Tab. 4: Combination of oxfendazole with interleukin-5 leads to absence of late embryonal stages**. 6 week old female BALB/c mice were naturally infected with *Litomosoides sigmodontis* and treated with 12.5 mg/kg oxfendazole twice per day for 5 days (positive control) or 3 days (shortened treatment) with or without addition of intranasal application of 2 µg IL-4, IL-5 or IL-33 once per day. Necropsies were performed 70 days after the infection. Embryonal development was analyzed in up to 5 intact female worms per mouse. Data for IL-4, IL-5, IL-33 from 1 experiment, data for other groups pooled from 2 experiments. Statistical analysis using Kruskal-Wallis with Dunn’s post hoc test, * p<0.05, ** p<0.01, *** p<0.001.

| Strain | Treatment | Oocyte | | Morula | | Pretzel | | Stretched MF | | Degenerated early | | Degenerated late | |
| --- | --- | --- | --- | --- | --- | --- | --- | --- | --- | --- | --- | --- | --- |
|  |  | **Mean ± SEM**  **stat. significance** | | **Mean ± SEM**  **stat. significance** | | **Mean ± SEM**  **stat. significance** | | **Mean ± SEM**  **stat. significance** | | **Mean ± SEM**  **stat. significance** | | **Mean ± SEM**  **stat. significance** | |
| BALB/c | **Vehicle** | 789.7  ± 149.6 |  | 323.4  ± 75.8 |  | 688.2  ± 230.6 |  | 854.5  ± 265.3 |  | 779.1  ± 116.3 |  | 221.4  ± 50.3 |  |
|  | **5d OXF** | 284.2  ± 94.3 | ns | 135.7  ± 49.3 | ns | 22.1  ± 15.4 | * | 0.7  ± 0.7 | ** | 337.1  ± 98.9 | ns | 10.7  ± 7.7 | * |
|  | **3d OXF** | 213.5  ± 38.0 | * | 98.3  ± 37.6 | ns | 137.1  ± 61.8 | ns | 36.7  ± 24.9 | * | 350.9  ± 76.4 | * | 21.9  ± 7.6 | ns |
|  | **OXF**  **+IL-4** | 246.6  ± 72.9 | * | 161.4  ± 65.8 | ns | 250.9  ± 91.8 | ns | 17.1  ± 8.2 | ns | 353.8  ± 101.4 | * | 14.7  ± 7.9 | ns |
|  | **OXF**  **+IL-5** | 370.0  ± 156.8 | ns | 250.0  ± 171.2 | ns | 5.8  ± 4.3 | ** | 0.0  ± 0.0 | ** | 583.3  ± 278.1 | ns | 0.0  ± 0.0 | ** |
|  | **OXF**  **+IL-33** | 408.7  ± 98.3 | ns | 146.6  ± 51.2 | ns | 48.3  ± 29.4 | ** | 4.1  ± 4.1 | *** | 359.1  ± 76.0 | ns | 9.5  ± 5.7 | ** |
|  | **IL-4** | 751.1  ± 220.3 | ns | 367.7  ± 143.7 | ns | 42.2  ± 26.8 | * | 122.2  ± 89.8 | ** | 1113.3  ± 268.7 | ns | 11.1  ± 7.6 | ** |
|  | **IL-5** | 376.6  ± 129.5 | ns | 234.0  ± 136.5 | ns | 280.0  ± 220.8 | * | 346.6  ± 312.7 | * | 339.3  ± 73.5 | ns | 127.3  ± 112.6 | ns |
|  | **IL-33** | 648.6  ± 185.1 | ns | 475.3  ± 260.0 | ns | 111.3  ± 55.8 | ns | 262.0  ± 177.5 | ns | 496.0  ± 153.5 | ns | 42.0  ± 23.3 | ns |
